# Community Analysis of Root- and Tuber-Associated Bacteria in Field-Grown Potato Plants Harboring Different Resistance Levels against Common Scab

**DOI:** 10.1264/jsme2.ME15109

**Published:** 2015-12-09

**Authors:** Akira Kobayashi, Yuki Ohdaira Kobayashi, Nobutaka Someya, Seishi Ikeda

**Affiliations:** 1 National Agricultural Research Center for Hokkaido Region, National Agriculture and Food Research Organization9–4 Shinsei-minami, Memuro-cho, Kasai-gun, Hokkaido 082–0081Japan

**Keywords:** 16S rRNA gene, common scab, community analysis, potato, rhizosphere

## Abstract

Eight genotypes of potato plants with different resistance levels against common scab were grown in a field infested with *Streptomyces turgidiscabies*. DNA was extracted from the roots, tubers, and rhizosphere soils of each of the eight genotypes at the flowering stage, and the quantity of *S. turgidiscabies* genomic DNA was assessed by real-time PCR using a TaqMan probe. The results obtained showed that the different potato genotypes had significant impacts on the population levels of *S. turgidiscabies* between resistant and susceptible genotypes in the tubers, but not in the roots or rhizosphere soils. Clone analyses of 16S rRNA gene libraries from the eight potato genotypes identified three phyla (*Proteobacteria*, *Firmicutes*, and *Actinobacteria*) as dominant taxa in root and tuber clone libraries, while a clustering analysis identified 391 operational taxonomic units (OTUs) at the species level. Eleven OTUs closely related to *Aquicella siphonis*, *Arthrobacter nicotinovorans*, *Streptomyces rishiriensis*, *Rhodococcus baikonurensis*, *Rhizobium radiobacter*, *Rhizobium etli*, *Phyllobacterium myrsinacearum*, *Paenibacillus pabuli*, *Paenibacillus alginolyticus*, and *Bacillus halmapalus* were detected in the root or tuber libraries of all the potato genotypes examined. Furthermore, an abundance of OTUs related to *Aquicella* and *Rhodococcus* was observed in the rhizospheres of resistant and susceptible potato genotypes, respectively. Based on this ecological information, an efficient survey may be conducted for biological agents from the potato rhizosphere.

Common scab of potato (*Solanum tuberosum* L.) is prevalent in most potato-growing areas around the world. It is caused by soil- or seed-borne pathogenic *Streptomyces*, including *Streptomyces scabies*, *S. acidiscabies*, *S. turgidiscabies*, *S. europaeiscabiei*, *S. stelliscabiei*, and other species (6, 24, 44, 49). When scab-like lesions are formed on tubers, their market value is significantly reduced. Several methods are currently used to control common scab, including the chemical treatment of seed potatoes (43), crop rotation (23, 45), incorporation of green manure (41, 45), reduction in soil pH (45), and irrigation during tuber formation (23, 29). However, the disease-suppressive effects of these methods are not sufficient. Strong reductions in the incidence of common scab may be achieved by soil fumigation with chemicals such as methyl bromide and chloropicrin; however, this treatment impairs the integrity of the ecosystem. Furthermore, these chemicals reduce not only the populations of pathogens, but also those of non-targeted microorganisms including beneficial soil microorganisms for disease control with a potential environmental risk and high cost. Therefore, alternative methods to control potato common scab are being widely sought.

Biological control is considered as a desirable approach for controlling soil-borne diseases because it is eco-friendly and expected to replace or supplement chemical pesticides (51). A number of antagonistic microorganisms including fungal species from the genus *Trichoderma* (19, 30); actinomycetes from the genus *Streptomyces* (2, 13, 18, 22, 50); general soil bacteria from the genera *Bacillus* (11, 28, 38); *Pseudomonas*, *Enterobacter*, and *Acinetobacter* (46); and bacteriophages (27) are known for their potential suitability as biocontrol agents for potato common scab. However, only limited success has been achieved in the development of a commercial application to control common scab using these microorganisms because of the inconsistent performance of biocontrol agents in field conditions, and, hence, this has become an important technical issue in the development and utilization of beneficial microorganisms in agronomic environments. The success of the smart utilization of beneficial microorganisms is now considered to largely depend on a comprehensive knowledge of plant-microbe interactions at a community level in field conditions (42). Thus, a better understanding of the diversity and functionality of the plant-associated microbial community in field conditions may facilitate the utilization of beneficial microorganisms in order to promote plant growth and control plant pathogens in agricultural practices (3).

Several studies using culture-independent methods have been reported for the characterization of the rhizosphere-associated bacterial community structures of potato plants (3, 9, 20, 35, 39, 40, 42). These culture-independent analyses not only revealed the tissue-specific distribution of potato-associated bacteria, but also contributed to the identification of candidate microorganisms for biological control (3, 35, 40, 47). However, the genetic background of host plants, even at the cultivar level, has been suggested to affect the diversity and functionality of the community structure of plant-associated microorganisms (31). The potential impact of host plant genotypes on the plant-associated microbial community may be significant in the case of the potato because its intragenetic diversity is generally considered to be large owing to polyploidy and self-incompatibility.

In order to achieve the successful biological control of plant diseases, including potato common scab, it is also important to understand the ecology and epidemiology of pathogens through the specific detection and quantification of pathogenic *Streptomyces* on potato tissue and in soil under field conditions. A TaqMan real-time PCR assay using virulence gene *nec1* (5), SYBR Green quantitative real-time PCR assay using the *txtAB* operon (33), and quantitative competitive quenching probe PCR assay using the *nec1* gene (26) have been used for the accurate detection and quantification of pathogenic *Streptomyces* in the potato. Although a good correlation has been reported between the presence of *nec1* and pathogenicity, some nonpathogenic *nec1*-positive strains and numerous pathogenic *nec1*-negative strains have been described (33). The *Streptomyces txtAB* operon encodes the non-ribosomal peptide synthetase required for phytotoxin thaxtomin A production and is essential for the pathogenicity of all known pathogens of potato common scab (12). Thus, the *txtAB* operon is regarded as an optimal marker for pathogenic *Streptomyces* for potato common scab, and, as such, the development of a TaqMan real-time PCR assay system using the *txtAB* operon is preferred for the highly accurate quantification of pathogenic *Streptomyces* for potato common scab in soil.

In the present study, we utilized a TaqMan real-time PCR assay system using the *txtAB* operon in order to monitor the population of *S. turgidiscabies* in the rhizospheres of potato plants grown in a field artificially infested with *S. turgidiscabies* and assess the impact of eight potato genotypes on the population of *S. turgidiscabies*. Furthermore, root- and tuber-associated bacterial communities were compared between the common scab-resistant and -susceptible genotypes of the potato using culture-independent techniques in order to obtain useful information for a better understanding of the ecology of potato common scab and an efficient survey of biological control agents.

## Materials and Methods

### Plant materials and sampling

Eight potato genotypes (seven cultivars: Yukirasha (17), Snow March (16), Star Ruby, Snowden, Irish Cobbler [Japanese name *Danshakuimo*], Toyoshiro, and Piruka, and an experimental line 02005-10) were used to quantify the populations of *S. turgidiscabies* in rhizospheres and assess the diversity of rhizosphere bacteria.

Disease severity in the eight potato genotypes against common scab was evaluated between 2004 and 2009 in an experimental field artificially infested with *S. turgidiscabies* at the Memuro Research Station, Hokkaido Agricultural Research Center (42°89.2′ N, 143°07.7′ E, 93 m a.s.l.) based on a previously reported method (17). After harvesting, the disease index of each tuber was determined by the percentage of the surface area with scab lesions: no lesions=0 points; 1–3%=1 point; 4–13%=2 points; 14–25%=3 points; more than 26%=4 points. Disease severity was calculated using the following formula (∑[score of the disease index×number of tubers]/[4×total number of tubers]×100). Based on these field evaluations, the resistance levels of the eight potato genotypes were categorized as highly resistant against (Yukirasha [R1] and 02005-10 [R2]), resistant against (Snow March [R3]), moderately resistant against (Star Ruby [M1] and Snowden [M2]), and susceptible to (Irish Cobbler [S1], Toyoshiro [S2], and Piruka [S3]) common scab (Table 1). These eight genotypes were planted on May 19, 2010 in an experimental field artificially infested with *S. turgidiscabies* at the Memuro Research Station, with single rows of 3.6 m in length and a plant spacing of 30 cm per genotype in triplicate. The field was dressed with a commercial fertilizer (60, 170, and 102 kg for N, P_2_O_5_, and K_2_O ha^−1^, respectively) as a side dressing for basal fertilization.

Nine healthy plants in each genotype were selected visually at random and sampled on July 7, 2010. After the root system of each potato plant was carefully sampled, the soil loosely attached to the roots was removed by gentle shaking. The soil tightly attached to the roots was immediately collected by firmly shaking the root system in a plastic bag as a rhizosphere soil. Each rhizosphere soil sample was passed through a sieve (2 mm in diameter) to remove large organic matter and was stored individually at −30°C.

Root systems were immediately transported on ice to a laboratory and were separated into roots and tubers. Each tissue was washed with tap water and sterilized water, and then stored individually at −30°C until used for DNA extraction. The general chemical characteristics of the field soil at the time of sampling were analyzed by the Tokachi Nokyoren Agricultural Research Institute (Obihiro, Japan). The soil type was classified as Andosol, a type of Typic Hapludand according to the U.S. Department of Agriculture soil taxonomy. The characteristics of the soil samples are shown in Table 2. Tubers in the corresponding field described above were also sampled on August 30, 2010 and disease severity in the tubers was evaluated as described previously (18).

### DNA extraction from rhizosphere soil, roots, tubers, and mycelia of *S. turgidiscabies*

Rhizosphere soil DNA was extracted in accordance with the method of Ikeda *et al.* (14) with slight modifications. Briefly, an aliquot (0.4 g) of a soil sample was suspended in 0.5 mL of DNA extraction buffer (500 mM Tris [pH 8.0], 100 mM EDTA, 100 mM NaCl, 2% SDS) and 0.5 mL of 300 mM sodium phosphate buffer (pH 8.0) with 100 μL of 20% skim milk (Difco Laboratories, New Jersey, USA) in a 2-mL screw-capped tube. After adding 0.5 g of Zirconia/Silica beads (0.1 mm in diameter) (Biospec Products, Bartlesville, USA), the tubes were processed in a Fastprep 24 (MP Biomedical, Santa Ana, USA) for 45 s at 5.5 s. The tube was then centrifuged for 1 min at 14,000×*g* at room temperature (RT), and the supernatant was transferred carefully into a new microtube and mixed with 0.2 volumes of 8 M potassium acetate by inversion. After centrifugation for 5 min at 16,000×*g* at RT, the supernatant was transferred into a fresh microtube and mixed with 0.6 volumes of isopropanol and 0.1 volumes of 3 M sodium acetate (pH 5.2) by inversion. The tube was incubated for 5 min at RT, and DNA was pelleted by centrifugation for 5 min at 16,000×*g* at RT. The pellet was washed with 70% ethanol and re-suspended in 100 μL of TE buffer (10 mM Tris and 1 mM EDTA, pH 8.0). The DNA sample obtained was purified using MagExtractor PCR & Gel Clean up (TOYOBO, Osaka, Japan) followed by a QIAquick PCR Purification Kit (QIAGEN, Tokyo, Japan) in accordance with the manufacturer’s protocol.

Approximately 20 g of roots for each plant was ground into powder in liquid nitrogen using a mortar and pestle and used individually to extract bacterial cells. The tubers (0.5–1.0 cm in diameter) collected from each of the nine plants were combined as one composite sample for each genotype owing to the limited amount of sample. Fifty grams of tubers for each genotype was ground into powder in liquid nitrogen with a mortar and pestle as a composite sample for each genotype due to the low yield of the amount of bacterial cells extracted from these samples, and this was used to extract bacterial cells. Root- or tuber-associated bacterial cells were extracted and purified using a bacterial cell enrichment method (15). Total DNA was extracted from each enriched bacterial cell sample using a previously reported DNA extraction method (14). Purified rhizosphere soil, root, and tuber DNA samples were quantified using a Qubit dsDNA BR assay kit (Life Technologies, Carlsbad, USA).

*S. turgidiscabies* strain 94-3 was grown at 28°C for 3 d in Tryptic Soy Broth (Difco Laboratories, Detroit, USA), and approximately 100–200 mg of mycelia were precipitated by centrifugation for 15 min at 16,000×*g* at 10°C. Mycelia were suspended in 0.5 mL of DNA extraction buffer and 0.5 mL of 300 mM sodium phosphate buffer (pH 8.0) with 0.75 g of Zirconia/Silica beads (0.1 mm in diameter) in a 2-mL screw-capped tube. DNA was then isolated from mycelia using the same method as that described for rhizosphere soil DNA extraction.

### Quantification of genomic DNA of *S. turgidiscabies* in the potato rhizosphere using a TaqMan real-time PCR assay system

Rhizosphere soil DNA, root-associated bacterial DNA, and tuber-associated bacterial DNA samples were used to quantify the genomic DNA of *S. turgidiscabies* in the rhizosphere of each potato genotype by a real-time PCR assay using a TaqMan probe. StreptF (5′-GCAGGACGCTCACCAGGTAGT-3′) and StreptR (5′-ACTTCGACACCGTTGTCCTCAA-3′) were used as the PCR primer pairs (33). The TaqMan probe txtAB (5′-FAM-CGACGGAAAGTACTGGAT-MGB-3′) was newly designed based on the *txtAB* gene sequence of *S. turgidiscabies* between the StreptF and StreptR primers. The specificity of the probe was confirmed using a BLAST homology search. Real-time PCR was performed in a 16-μL reaction mixture containing 1×TaqMan Universal Master Mix II (Applied Biosystems, Tokyo, Japan), 0.9 μM of each primer, and 0.25 μM of TaqMan probe. A total of 1.6 μL of the rhizosphere soil, root, or tuber DNA extract was used as template DNA. Real-time PCR amplification was performed using a 7500 Fast Real-Time PCR System (Applied Biosystems) in accordance with the manufacturer’s protocol with an initial activation step at 95°C for 20 s, followed by 40 cycles of 95°C for 3 s and 60°C for 30 s. The amount of genomic DNA of *S. turgidiscabies* in a sample was calculated by referring the Ct value to a standard curve of Ct values based on the genomic DNA of *S. turgidiscabies* with serial dilutions ranging from 64 pg to 20 fg. Each DNA sample was subjected to the TaqMan real-time PCR assay system three times as pseudoreplicates, and the amount of genomic DNA of *S. turgidiscabies* was expressed as an average of the pseudoreplicates in order to obtain reliable results. Significance was tested for differences in the amount of genomic DNA of *S. turgidiscabies* in tubers between resistant and susceptible genotypes by the two sample *t*-test and among the eight potato genotypes by Tukey’s test with SPSS software (IBM, Tokyo, Japan). Differences in the amount of genomic DNA in the root and rhizosphere soil DNA samples of *S. turgidiscabies* among potato genotypes were examined using Tukey’s test (*P*<0.05 for significance).

### Clone library construction and sequencing

PCR clone libraries for 16S rRNA genes were constructed as described by Someya *et al.* (42). Briefly, PCR was performed to amplify 16S rRNA gene sequences using total DNA as a template. The universal primers 27F (5′-AGAGTTTGATCMTGGCTCAG-3′) and 1525R (5′-AAGGAGGTGWTCCARCC-3′) were used (21). PCR cycling was preceded by an initial 2-min denaturation at 94°C, and the reaction was then conducted for 25 cycles at 94°C for 30 s, at 55°C for 30 s, and at 72°C for 2 min, followed by the final extension of an incubation at 72°C for 7 min. The products of PCR derived from the roots of nine plants for each potato genotype were combined into a composite sample. The PCR products from the root- and tuber-associated bacteria were separated on a 1% agarose gel in 0.5×TBE buffer (89 mM Tris-Borate, 0.2 mM EDTA) and purified using NucleoSpin Extract II (Macherey-Nagel, Düren, Germany). DNA fragments were cloned into a pGEM-T Easy vector (Promega, Tokyo, Japan) and a clone library was constructed. Sequencing of the 16S rRNA genes of clone libraries was conducted by the Takara Bio Dragon Genomic Center (Takara Bio, Yokkaichi, Japan) using the 27F primer, as previously described (42).

### Sequence editing and analyses

Sequence editing and analyses were performed as described by Someya *et al.* (42). Approximately 500 bp of the 16S rRNA gene (corresponding to 109–665 bp of the *Escherichia coli* 16S rRNA gene) were used for sequence analyses. Briefly, 16S rRNA gene sequences were aligned using ClustalW, and a distance matrix was constructed using the DNAdist program from PHYLIP ver. 3.66 with default parameters and then analyzed by Mothur (37). Operational taxonomic units (OTUs) were defined with ≥97% identity for clustering analyses. Library coverage was calculated using the non-parametric estimator *C* (10). The reciprocal of Simpson’s index (1/*D*) was used as a measure that characterizes species diversity in a community (52). Similarities between clone libraries were determined by UniFrac (25). A principal coordinates analysis (PCoA) was performed using UniFrac with the abundance-weighted option. The phylogenetic composition of each clone library was evaluated using the LibCompare program of RDP-II release 10 (48), with confidence levels of 80%. A survey of the closest known species for clones in public databases was performed using BLASTN (1). The phylogenetic tree file was constructed by the neighbor-joining method (36) using the bootstrapping procedure (7), and the tree was drawn using TreeView software (32).

### Nucleotide sequence accession numbers

The nucleotide sequences analyzed in the present study were deposited in the DDBJ/EMBL/GenBank database. The sequence data of root and tuber clone libraries were deposited under accession numbers LC022796 to LC024137 and LC024138 to LC024802, respectively.

## Results

### Disease severity of eight potato genotypes

Eight potato genotypes were grown for 3 months until the foliage died back. After tubers had been harvested, disease severity was assessed in the eight potato genotypes. The disease severities of Yukirasha (R1), 02005-10 (R2), Snow March (R3), Star Ruby (M1), Snowden (M2), Irish Cobbler (S1), Toyoshiro (S2), and Piruka (S3) were 0.23, 0.23, 0.53, 5.1, 3.8, 28.3, 31.6, and 35.5, respectively. These values were similar to the known resistance levels of each genotype examined between 2004 and 2009.

### Quantification of genomic DNA of *S. turgidiscabies* in potato rhizospheres

**Fig. 1:**
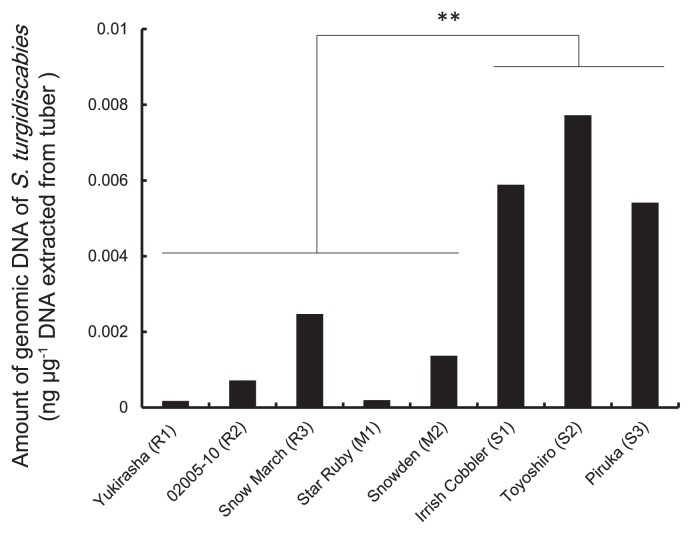
Quantification of genomic DNA of *Streptomyces turgidiscabies* in total DNA extracted from tubers. Eight genotypes of potato plants were grown in a field infested with *S. turgidiscabies*. DNA was extracted from the tubers of each of the eight genotypes at the flowering stage, and the quantity of genomic DNA of *S. turgidiscabies* was assessed by a real-time PCR assay using a TaqMan probe. The significance of differences in the amount of the genomic DNA of *S. turgidiscabies* between resistant and susceptible potato genotypes was tested by the two sample *t*-test. ** indicates significance at the 1% level (*P*<0.01).

In order to determine the detection limit and quantification range of the genomic DNA of *S. turgidiscabies*, the TaqMan real-time PCR assay using the primer pair StrepF/R and probe txtAB was performed using serial dilutions of the genomic DNA of *S. turgidiscabies*. A standard curve was constructed based on a PCR assay of three pseudoreplicates (Fig. S1). The standard curve showed a linear response with a high correlation coefficient (R^2^=0.9899). The efficiency of the real-time PCR amplification was 95%. The detection limit was approximately 12.8 fg. Therefore, these results confirmed the applicability of the TaqMan real-time PCR assay to accurately quantify *S. turgidiscabies*. Quantification of the genomic DNA of *S. turgidiscabies* in potato rhizospheres was conducted using a TaqMan real-time PCR assay system. Quantification of the genomic DNA of *S. turgidiscabies* in tuber DNA was shown to be positive for all eight potato genotypes, ranging from 0.2 to 7.7 pg in 1 μg of total DNA extracted from tubers (Fig. 1). A marked difference was observed in the genomic DNA of *S. turgidiscabies* in tuber DNA between resistant (R1, R2, R3, M1, and M2) and susceptible (S1, S2 and S3) genotypes against common scab (Fig. 1). The amount of genomic DNA of *S. turgidiscabies* in tubers was at least 41-fold higher in the susceptible genotype Toyoshiro (S2) than in the highly resistant genotype Yukirasha (R1). In addition, significant differences in the amount of genomic DNA of *S. turgidiscabies* in tuber DNA were detected at the genotype level between the susceptible and resistant groups. Quantification of the genomic DNA of *S. turgidiscabies* in the root or rhizosphere DNA of eight potato genotypes was also shown to be positive for all samples (Fig. S2). However, unlike tuber DNA samples, a significant difference was not observed between resistant (R1, R2, R3, M1, and M2) and susceptible (S1, S2, and S3) genotypes against common scab in root or rhizosphere DNA samples.

### Statistical analyses of clone libraries and isolate collections

**Fig. 2:**
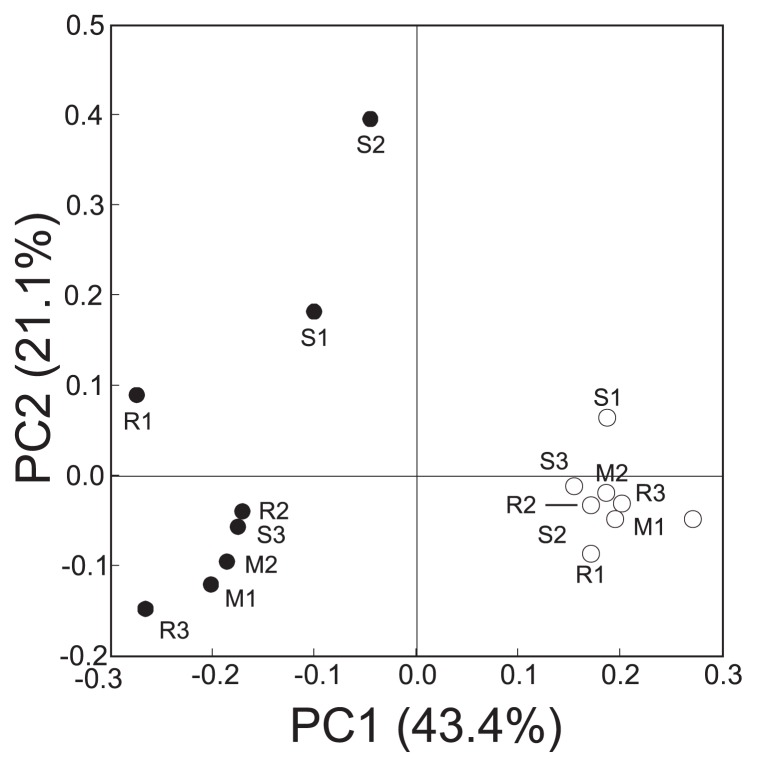
Principal-coordinates analysis of 16S rRNA gene sequences of clone libraries for bacteria derived from roots (○) and tubers (●) of eight potato genotypes (R1 [Yukirasha], R2 [02005-10], R3 [Snow March], M1 [Star Ruby], M2 [Snowden], S1 [Irish Cobbler], S2 [Toyoshiro], and S3 [Piruka]) grown in a field infested with common scab. The ordinations were constructed using UniFrac distances weighted by the relative abundance.

The statistical characteristics of the clone libraries are summarized in Table 3. Regardless of the resistance levels of the potato genotypes, the number of OTUs and diversity indexes were higher for root-associated bacteria than for the corresponding tuber-associated bacteria. Although a tuber and root are physically present close together in the soil, strong selectivity was shown to be present for bacterial diversity in tubers. The diversity of root-associated bacteria was smaller in resistant genotypes than in susceptible genotypes, as shown by the number of OTUs and Shannon and Simpson indexes. By analyzing the combined sequence data set from all clone libraries in the present study, 97 genera in eight phyla and 391 OTUs (clustering with ≥97% identity) were identified across the rhizospheres of the eight genotypes examined (Tables S1 and S2). The results of PCoA revealed the presence of distinct community structures between root-and tuber-associated bacteria, as indicated by the community shifts along PC1 (43.4%) (Fig. 2). In roots, the bacterial community structures were highly similar among the eight genotypes, as shown by the tight clustering. In contrast, the bacterial community structures in tubers appeared to be more variable than those in roots among the eight potato genotypes, as shown by the loose clustering along PC2 (21.1%) regardless of the resistance levels of potato genotypes.

### Phylogenetic analyses of clone libraries

Analyses of phylogenetic compositions using LibCompare of RDP II revealed that clone libraries were exclusively dominated by three or four phyla (Table 4). Three phyla (*Proteobacteria*, *Firmicutes*, and *Actinobacteria*) were found as the main taxa in root and tuber clone libraries (Table 4). The dominant phylum among root libraries was *Proteobacteria* (57.7–73.5%). It was also the dominant phylum in the tuber libraries of Yukirasha (57.8%), 02005-10 (60.4%), Irish Cobbler (51.1%), and Toyoshiro (74.1%). *Firmicutes* was the dominant phylum in the tuber libraries of Snow March (69.8%), Star Ruby (59.8%), Snowden (50.7%), and Piruka (41.1%).

**Fig. 3:**
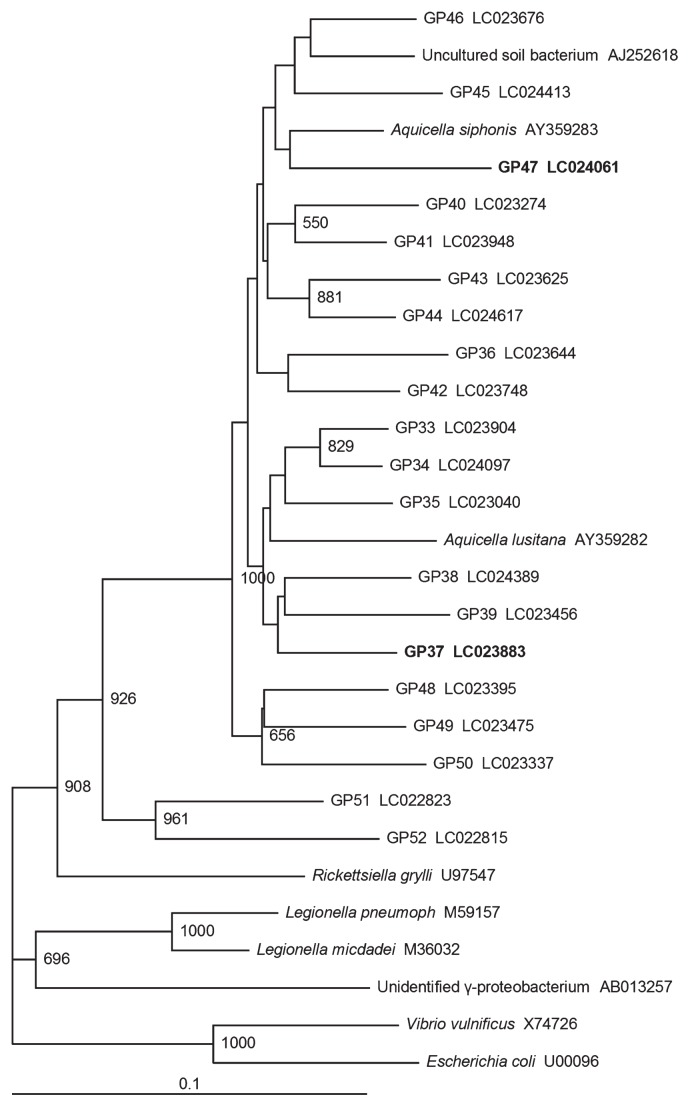
Phylogenetic tree of representative sequences of OTUs for novel *Gammaproteobacteria* related to the genus *Aquicella* in the potato rhizosphere. The tree was constructed by the neighbor-joining method. The scale represents 0.1 substitutions per site. The numbers at the nodes are the proportions of 1,000 bootstrap resamplings; however, values <500 are not shown. The representative sequences of two OTUs (GP37 and GP47) discussed in the main text are indicated in bold.

Within *Proteobacteria*, *Gammaproteobacteria* was identified as the dominant group in root clone libraries (19.4–37.0%) (Table 4) and 124 OTUs were observed for *Gammaproteobacteria* by a clustering analysis (Table S2). In contrast, *Gammaproteobacteria* were less abundant in the tuber libraries (2.5–15.8%), with only 18 OTUs being identified. Only two genera (*Aquicella* and *Pseudomonas*) were shown to be major taxa at the genus level of *Gammaproteobacteria* in the root clone libraries (6.8–18.6% and 0–3.5%, respectively, in Table 4). The relative abundance of the genus *Aquicella* in the root clone library was significantly higher for Yukirasha (R1) than for Snow March (R3), Snowden (M2), Irish Cobbler (S1), Toyoshiro (S2), and Piruka (S3) (Table 4 and Table S3 A). The relative abundance of *Aquicella* in the root clone library was also significantly higher for 02005-10 (R2) than for Snowden (M2), Toyoshiro (S2), and Piruka (S3) (*P*<0.05) (Table S3 A). Thus, *Aquicella* clones were slightly more dominant in the root libraries of highly resistant genotypes than those in other genotypes. Clustering analyses identified two abundant OTUs (GP37 and GP47) related to *Aquicella* in the highly resistant genotypes Yukirasha (R1) and 02005-10 (R2), respectively (Table S2). The representative sequences of these two OTUs showed only 92% identity with *Aquicella siphonis*. Based on a phylogenetic tree analysis of the OTUs showing the highest identities to *A. siphonis*, marked sequence variations were found to be present among these OTUs (Fig. 3).

*Alphaproteobacteria* was dominant in root and tuber clone libraries (13.5–36.6% and 18.6–65.4%, respectively) (Table 4), with 65 OTUs being identified in all clone libraries (Table S2). The genus *Phyllobacterium* was mainly responsible for the dominance of *Alphaproteobacteria* in tubers. An OTU in *Phyllobacterium* sp. (OTU AP39 in Table S2) was shown to be present in all tuber clone libraries. Although the genus *Rhizobium* was responsible for the dominance of *Alphaproteobacteria* in roots (Table 4), and two OTUs (AP27 and AP31; Table S2) were detected in all root clone libraries. The representative sequences of these two OTUs showed 100% and 99% identities to *Rhizobium radiobacter* and *Rhizobium etli*, respectively.

In *Firmicutes*, the genus *Paenibacillus* was found to be abundant in all root libraries (8.1–18.6%) (Table 4). In this genus, two OTUs (FM12 and FM20) were highly abundant in all root libraries (Table S2). The representative sequences of these OTUs showed 100% and 99% identities to *Paenibacillus pabuli* and *Paenibacillus alginolyticus*, respectively. In contrast, the genus *Bacillus* was dominant in all tuber libraries (8.6–58.1%). Clustering analyses revealed that most of the clones for this genus in tubers belonged to OTU FM41 (7.4– 60.5% in Table S2). The representative sequence of OTU FM41 was identical to that of *Bacillus halmapalus*.

In *Actinobacteria*, the genus *Rhodococcus* was more abundant in the tubers of susceptible genotypes than in those of resistant genotypes (Table 4 and Table S3 B). Most clones belonging to this genus were clustered into OTU AC24, with 100% identity to *Rhodococcus baikonurensis* (Table S2). Meanwhile, two genera, *Arthrobacter* and *Streptomyces*, were found in most root libraries (Table 4), and the corresponding dominant OTUs were identified (AC5 and AC16, respectively; Table S2). The representative sequences of these OTUs showed 100% and 99% identities to *Arthrobacter nicotinovorans* and *Streptomyces rishiriensis*, respectively. Although *S. turgidiscabies* was detected in DNA samples extracted from roots and tubers by real-time PCR (Fig. 1 and Fig. S2), the OTU corresponding to this species was not found in the root or tuber libraries examined in the present study.

## Discussion

A TaqMan real-time PCR assay using primers (33) and a newly designed probe (present study) based on the *txtAB* operon was developed in the present study for the specific detection of the pathogenic *Streptomyces* species responsible for potato common scab. Multiplex TaqMan real-time PCR assays for the simultaneous detection and discrimination of potato powdery and common scab diseases and their pathogens have been reported in recent years (34). The sequence of the TaqMan probe txtAB designed in the present study differed from that of the probe StrepP previously designed by Qu *et al.* (34). The detection limit of the TaqMan real-time PCR assay developed in the present study was approximately 12.8 fg of the genomic DNA of *S. turgidiscabies* (Fig. S1), which was of equal sensitivity to 10 fg of *Streptomyces* DNA in the SYBR Green quantitative real-time PCR assay using the *txtAB* operon (33). Thus, we concluded that the PCR assay developed in the present study was a sensitive and specific tool for the detection of common scab pathogens.

Eight potato genotypes showing different resistance levels to common scab (Table 1) were grown in a field artificially infested with *S. turgidiscabies*, and the colonization of potato tubers, roots, and rhizosphere soil at the flowering stage was quantitatively analyzed using the TaqMan real-time PCR assay described above. The results obtained revealed significant differences in the population levels of *S. turgidiscabies* in tubers at the flowering stage between resistant (R1, R2, R3, M1, and M2) and susceptible (S1, S2, and S3) potato genotypes (Fig. 1). Disease severities in the tubers of the eight potato genotypes at the harvest stage almost corresponded with the amounts of genomic DNA of *S. turgidiscabies* in tubers at the flowering stage when apparent disease symptoms were not observed in the tubers. Therefore, the evaluation of potato genetic materials for potato common scab appears to be possible by quantifying the population of pathogens in tubers in the early growing period of potato plants. This novel method may contribute to facilitating the effective screening of potato genetic resources for scab resistance in a breeding program and developing rapid surveying methods for biocontrol agents in order to suppress common scab disease. In contrast to tubers, the amount of *S. turgidiscabies* genomic DNA in the roots and rhizosphere soil did not correlate with the resistance level of the potato genotype against potato common scab (Fig. S2). These results suggest that the resistance level of a potato genotype to common scab did not significantly influence the colonization levels of *S. turgidiscabies* in the roots and rhizosphere soil at the flowering stage.

Analyses of the phylogenetic compositions of clone libraries revealed that potato-associated bacterial communities were dominated by only a few phyla, mainly consisting of *Proteobacteria*, *Firmicutes*, *Actinobacteria*, and *Planctomyces* (Table 4), as shown in a previous study (42). *Gammaproteobacteria*, *Alphaproteobacteria*, and *Actinobacteria* appeared to be stable and the dominant bacterial groups in roots and tubers across the eight potato genotypes examined.

Phylogenetic analyses also revealed the presence of a phylogenetically novel gammaproteobacterial group showing very low identity to *A. siphonis* as one of the dominant groups in potato roots in the present study (Table 4 and Table S2), which was consistent with our previous findings (42). The abundance of this novel gammaproteobacterial group was shown to be higher in the two genotypes of the potato that were highly resistant to common scab (Yukirasha and 02005- 10) than in the other genotypes (Table S3 A). The abundance of OTU GP37 was shown to be significantly higher in the roots of the highly resistant genotype Yukirasha (R1). The abundance of OTU GP47 was also found to be high in the roots of the highly resistant genotype 02005-10 (R2). Although the antagonistic potential of these OTUs against potato common scab currently remains unknown because of the inability to culture them, the abundance of these two OTUs may be used as an index species to select a resistant potato genotype against common scab in the absence of pathogens. Further studies are needed in order to determine whether the abundance of these phylogenetically novel gammaproteobacterial groups correlates with scab resistance to some extent in other potato genotypes. In contrast to these unknown *Gammaproteobacteria*, the relative abundance of *Rhizobium* sp. was shown to be relatively low in the two highly resistant potato genotypes (Table 4), implying competition for a niche between *Aquicella* and *Rhizobium* genera.

*Actinomycetes* are known for their potential suitability as biocontrol agents for potato common scab (2, 13, 18, 22, 50). Three dominant OTUs (AC5, AC16, and AC24) with high identities to *A. nicotinovorans*, *S. rishiriensis*, and *R. baikonurensis* were identified in the roots of most of genotypes examined (Table S2). The relative abundance of OTU AC24 was higher in the tubers of susceptible genotypes than in those of resistant genotypes (Table S2). OTU AC24 may play a role in infections by potato common scab, and its high abundance in tubers may be used as an indicator to select potato genotypes against common scab in the absence of pathogens. Plant-associated *Rhodococcus* spp. is known as a producer of 1-aminocyclopropane-1-carboxylic acid (ACC) deaminase by utilizing ACC as a nitrogen source (8). When the potato is infected with common scab, ethylene production may be promoted by the host and pathogens (4), and this may enhance the abundance of *Rhodococcus* spp. in the tubers of susceptible genotypes. Regarding the genus *Aquicella*, it will be interesting to determine whether the abundance of OTU AC24 correlates with susceptibility to common scab to some extent in other potato genotypes. Since *Arthrobacter* and *Streptomyces* are both known for their antagonistic activities against soil-borne diseases in the potato (8), bacterial isolates belonging to OTUs AC5 and AC16 may be primary candidates as biocontrol agents against potato common scab.

Two genera, *Paenibacillus* and *Bacillus*, were exclusively detected in the root and tuber clone libraries, respectively (Table S1). *Paenibacillus* sp. is known to exhibit antagonistic activity against several pathogens of the potato (40). Several bacteria from the genus *Bacillus* (11, 28, 38) are also known for their potential suitability as biocontrol agents, and a species in this genus has been commercialized in Japan as a control agent for common scab (18). The high stability of *Paenibacillus* and *Bacillus* in the potato rhizosphere suggests that they are suitable candidates for controlling common scab from an ecological viewpoint. *Paenibacillus* and *Bacillus* are both spore-forming bacteria that are highly tolerant to dryness as Gram-positive bacteria. These properties of *Paenibacillus* and *Bacillus* indicate that they are a resourceful group for the screening of practical biocontrol agents against potato common scab.

In summary, the present study revealed that potato genotypes against common scab strongly impacted on the population levels of *S. turgidiscabies* in tubers. A total of 391 OTUs were identified at the species level in the present study. Of these, 11 OTUs related to *A. siphonis* (OTUs GP37 and GP47), *A. nicotinovorans* (OTU AC5), *S. rishiriensis* (OTU AC16), *R. baikonurensis* (OTU AC24), *R. radiobacter* (OTU AP27), *R. etli* (OTU AP31), *P. myrsinacearum* (OTU AP39), *P. pabuli* (OTU FM12), *P. alginolyticus* (OTU FM20), and *B. halmapalus* (OTU FM41) were found at appreciable levels in the root or tuber libraries of all the potato genotypes examined. Based on these ecological data, it may be possible to conduct an efficient survey for biological agents from the potato rhizosphere. Furthermore, the high abundance of some OTUs related to *Aquicella* and *Rhodococcus* in the rhizospheres of resistant and susceptible potato genotypes, respectively, may be a useful microbial indicator for evaluating the resistance level of a potato genotype against common scab in the absence of pathogens. However, these results need to be reexamined in an uninfested field and also in a field with different soil types or geological locations in future studies. A spatial investigation also needs to be conducted in order to determine the impact of potato genotypes on the microbial community in rhizosphere soil. The results of the present study suggest that the unstable performances of biocontrol agents in field conditions in previous studies were due to distinct differences between laboratory and field conditions in terms of the ecological behavior of plant-associated microorganisms that depended on their host plant genotypes and diverse environmental factors.

## Supplementary Material



## Figures and Tables

**Table 1:** Characteristics of 8 potato genotypes harboring different resistance levles against common scab.

Cultivers/line	Abbreviation	Resistance levels against common scab	Cross parent		Maturity	Usage

			female	male		
Yukirasha	R1	Highly resistant	Early Gem	86002-100	medium-early	table use
02005-10	R2	Highly resistant	Yukirahsa	Pike	medium	table use
Snow March	R3	resistant	Atlantic	Cherokee	medium	table use
Star Ruby	M1	moderately resistant	Hokkai No. 77	87028-6	medium	table use
Snowden	M2	moderately resistant	B5141-6	Wischip	medium-late	chips
Irish Cobbler	S1	susceptible	unknown	unknown	early	table use
Toyoshiro	S2	susceptible	Hokkai No. 19	Eniwa	medium-early	chips
Piruka	S3	susceptible	Meihou	Tokachikogane	medium-early	table use

**Table 2:** Chemical characteristics of soil at the time of sampling rhizospheres.

pH (H_2_O)	Organic C (g kg^−1^)	Total N (g kg^−1^)	Available N (mg kg^−1^)	NH_4_-N (mg kg^−1^)	NO_3_-N (mg kg^−1^)	Truog P (mg kg^−1^)	Ex-K_2_O (mg kg^−1^)	Ex-CaO (mg kg^−1^)	Ex-MgO (mg kg^−1^)	CEC (meq kg^−1^)	PAC^a^
5.9	29	2.5	73.4	10.3	9.3	105	365	1830	544	185	1639

a phosphate absorption coefficient.

**Table 3:** Statistical characteristics of clone libraries of 16S rRNA gene sequences for root- and tuber-associated bacteria of 8 potato genotypes.

Statistics and diversity indexes	Clone libraries															

	Root								Tuber							
	
	R1^a^	R2	R3	M1	M2	S1	S2	S3	R1	R2	R3	M1	M2	S1	S2	S3
No. of sequences	156	165	173	162	170	175	164	177	135	53	43	97	69	92	81	95
No. of singletons	50	47	43	44	40	60	68	51	20	17	10	31	21	25	16	18
Library coverage (%)^b^	67.9	71.5	75.1	72.8	76.5	65.7	58.5	71.2	85.2	67.9	76.7	68.0	69.6	72.8	80.2	81.1
No. of OTUs^c^	71	75	70	71	74	86	93	81	27	22	14	41	31	32	24	29
Chao1	224.1	152.2	152.1	138.6	119.9	246.9	244.9	187.3	65.0	67.3	29.0	118.5	73.0	182.0	54.0	59.6
ACE	456.2	264.4	245.7	231.5	175.6	438.3	541.7	306.1	198.4	382.7	36.5	301.6	107.1	300.1	105.7	140.3
Shannon (*H*′)	3.8	3.9	3.8	3.8	4.0	4.0	4.2	4.0	1.8	2.5	1.7	2.9	2.9	2.6	2.2	2.6
Simpson (1/*D*)	31.5	31.4	34.1	32.3	47.3	40.2	56.2	46.1	3.5	8.2	2.7	7.2	11.1	7.5	4.1	8.5

a R, M, and S indicate the phenotypes of the 8 potato genotypes: resistant, medium resistant, and susceptible to potato scab diseases, respectively. R1, R2, R3, M1, M2, S1, S2, and S3 are designated for clone libraries of the 8 potato genotypes (Yukirasha, 02005-10, Snow March, Star Ruby, Snowden, Irish Cobbler, Toyoshiro, and Piruka, respectively).

b *C_X_*=1 (*n*/*N*), where *n_x_* is the number of singletons that are encountered only once in a library and *N* is the total number of clones.

c OTUs were defined at ≥97% sequence identity.

**Table 4:** Phyllogenetic compositions of major taxa for root- and tuber-assoicated bacteria in 8 potato genotypes.

Phyllogenetic composition^a^	Clone libraries (%)															

	Root								Tuber							
	
	R1^b^	R2	R3	M1	M2	S1	S2	S3	R1	R2	R3	M1	M2	S1	S2	S3
Actinobacteria	11.5	9.1	8.7	3.7**	11.8	8.6	6.1	9.6	3.0	3.8	2.3	10.3	8.7	15.2**	13.6**	23.2**
*Rhodococcus*	1.9	3.0	2.9	—	3.5	4.0	1.2	1.7	1.5	1.9	—	—	1.4	12.0**	8.6*	20.0**
*Streptomyces*	3.8	1.2	2.3	—*	4.1	0.6*	1.2	1.1	—	—	2.3	—	1.4	1.1	1.2	—
*Arthrobacter*	1.9	3.0	1.7	1.9	2.4	1.7	1.8	1.7	0.7	—	—	1.0	—	1.1	1.2	2.1
Bacteroidetes	—	—	—	—	1.8	—	—	—	—	—	—	—	—	—	—	—
*Flavobacterium*	—	—	—	—	1.8	—	—	—	—	—	—	—	—	—	—	—
Chloroflexi	0.6	—	—	0.6	—	0.6	—	—	—	—	—	—	—	—	—	—
Firmicutes	21.8	18.2	12.7*	19.8	13.5	10.3**	18.9	15.8	38.5	35.8	69.8**	59.8**	50.7	26.1	9.9**	41.1
Clostridia	—	—	—	0.6	0.6	—	—	—	0.7	—	7.0	4.1	1.4	1.1	—	—
Bacilli	21.8	18.2	12.7*	19.2	12.9*	10.3**	18.9	15.8	36.3	35.8	60.5**	54.6**	49.3	25.0	8.6**	37.9
*Paenibacillus*	18.6	12.1	8.1**	16.7	12.9	8.6**	12.2	10.2*	3.0	3.8	—	3.1	13.0**	4.3	—	3.2
*Bacillus*	2.6	5.5	4.6	2.5	—	1.7	5.5	5.1	32.6	30.2	58.1**	40.2	29.0	18.5*	8.6**	32.6
Gemmatimonadetes	—	—	—	—	—	0.6	—	—	—	—	—	—	—	—	—	—
Planctomycetes	5.1	2.4	3.5	2.5	4.7	9.7	4.9	1.7	—	—	—	1.0	—	3.3	2.5	—
*Zavarzinella*	1.9	0.6	0.6	0.6	0.6	1.7	0.6	0.6	—	—	—	—	—	2.2	2.5	—
Proteobacteria	57.7	67.3	72.8**	73.5**	64.7	62.9	64.0	68.4	57.8	60.4	23.3**	27.8**	40.6*	51.1	74.1*	35.8**
Alphaproteobacteria	13.5	21.2	26.6**	27.2**	28.2**	36.6**	18.9	32.8**	49.6	45.3	18.6**	19.6**	31.9*	43.5	65.4*	18.9**
*Phyllobacterium*	—	2.4	—	—	—	—	0.6	—	44.4	13.2**	7.0**	7.2**	7.2**	30.4*	48.1	1.1**
*Rhizobium*	2.6	6.7	13.3**	14.8**	8.2*	21.1**	7.9*	16.4**	2.2	1.9	4.7	—	4.3	8.7*	9.9*	5.3
*Devosia*	1.9	1.2	1.7	1.9	2.4	1.1	1.2	2.3	—	—	—	—	1.4	—	1.2	—
Betaproteobacteria	3.2	10.3*	3.5	7.4	5.9	5.7	8.5	5.6	1.5	5.7	—	1.0	—	4.3	4.9	1.1
Gammaproteobacteria	33.3	35.2	37.0	37.0	28.2	19.4**	27.4	24.3	5.9	7.6	4.7	6.2	7.2	3.3	2.5	15.8*
*Aquicella*	18.6	15.8	9.2*	13.0	7.1**	9.1*	7.9**	6.8**	—	1.9	—	3.1	—	1.1	—	—
*Pseudomonas*	—	2.4	3.5*	2.5	1.2	1.1	1.2	2.8*	0.7	5.7*	4.7	—	4.3	—	1.2	4.2
Unclassified																
Gammaproteobacteria	12.8	9.7	16.8	9.3	4.1	5.1	11.0	5.1	—	—	—	1.0	1.4	1.1	1.2	1.1
Deltaproteobacteria	6.4	0.6**	5.8	1.2*	1.2*	1.1*	7.3	5.6	—	1.9	—	1.0	—	—	—	—
Verrucomicrobia	1.3	1.8	1.2	0.0	3.5	5.7*	3.7	4.0	—	—	—	—	—	2.2	—	—
Bacteria_incertae_sedis	—	—	—	0.6	1.8	0.6	—	—	—	—	—	—	—	—	—	—
Unclassified Bacteria	1.9	1.2	1.2	—	—	1.7	2.4	0.6	0.7	—	4.7	1.0	—	2.2	—	—

a Sequences were grouped using the RDP Classifier of the Ribosomal Database Project-II release 11 with a confidence threshold of 80%.

b R, M, and S indicate the phenotypes of the 8 potato genotypes; resistant, medium resistant, and susceptible to potato scab diseases, respectively. R1, R2, R3, M1, M2, S1, S2, and S3 are designated for clone libraries of the 8 potato genotypes (Yukirasha, 02005-10, Snow March, Star Ruby, Snowden, Irish Cobbler, Toyoshiro, and Piruka, respectively). CD stands for the isolate collections derived from roots or tubers of cultivar “Yukirasha”.

* and ** indicate significance at the 5% and 1% levels (*P*<0.05 and *P*<0.01), respectively, calculated with the Library Compare of RDP II, between R1 and other libraries.
