# Supplementary material for: Community Analysis of Root- and Tuber-Associated Bacteria in Field-Grown Potato Plants Harboring Different Resistance Levels against Common Scab
Source: Microbes Environ. 2015 Dec 9;30(4):301–9. doi: 10.1264/jsme2.ME15109 (PMC4676553; doi:10.1264/jsme2.ME15109)
Supplement: Supplementary file 1 [file 30_301_s1.pdf]

Supplementary Table 1. Phyllogenetic compositions of clone libraries of 16S rRNA gene sequences for root- and tuber-associated bacteria of 8 potato genotypes

Phyllogenetic composition[a]	Clone libraries															
	Root								Tuber							
	R1[b]	R2	R3	M1	M2	S1	S2	S3	R1	R2	R3	M1	M2	S1	S2	S3
Actinobacteria	11.5	9.1	8.7	3.7**	11.8	8.6	6.1	9.6	3.0	3.8	2.3	10.3	8.7	15.2**	13.6**	23.2**
Actinobacteria	11.5	9.1	8.7	3.7**	11.8	8.6	6.1	9.6	3.0	3.8	2.3	10.3	8.7	15.2**	13.6**	23.2**
Actinobacteridae	11.5	9.1	8.7	3.7**	11.8	8.6	6.1	9.6	3.0	3.8	2.3	10.3	8.7	15.2**	13.6**	23.2**
Actinomycetales	11.5	9.1	8.1	3.7**	11.8	8.6	6.1	9.0	3.0	3.8	2.3	9.3	8.7	15.2**	13.6**	23.2**
Corynebacterineae	4.5	3.0	3.5	0.6*	3.5	4.0	1.8	2.3	1.5	3.8	-	5.2	7.2	13.0**	9.9**	21.1**
Mycobacteriaceae	2.6	-	0.6	-	-	-*	-	0.6	-	1.9	-	5.2	-	1.1	-	1.1
Mycobacterium	2.6	-	0.6	-	-	-*	-	0.6	-	1.9	-	5.2	-	1.1	-	1.1
Nocardiaceae	1.9	3.0	2.9	0.6	3.5	4.0	1.8	1.7	1.5	1.9	-	-	7.2	12.0**	8.6*	20.0**
Rhodococcus	1.9	3.0	2.9	-	3.5	4.0	1.2	1.7	1.5	1.9	-	-	1.4	12.0**	8.6*	20.0**
Nocardia	-	-	-	0.6	-	-	0.6	-	-	-	-	-	-	-	-	-
Smaragdicoccus	-	-	-	-	-	1.1	-	-	-	-	-	-	-	-	-	-
Unclassified Corynebacterineae	-	-	-	-	-	-	-	-	-	-	-	-	-	-	1.2	-
Streptomycineae	3.8	1.8	2.3	0.6	4.1	0.6*	1.8	1.1	-	-	2.3	-	1.4	1.1	1.2	-
Streptomycetaceae	3.8	1.8	2.3	0.6	4.1	0.6*	1.8	1.1	-	-	2.3	-	1.4	1.1	1.2	-
Streptomyces	3.8	1.2	2.3	-*	4.1	0.6*	1.2	1.1	-	-	2.3	-	1.4	1.1	1.2	-
Kitasatospora	-	0.6	-	0.6	-	-	-	-	-	-	-	-	-	-	-	-
Unclassified Streptomycetaceae	-	-	-	-	-	-	0.6	-	-	-	-	-	-	-	-	-
Propionibacterineae	0.6	-	0.6	-	-	0.6	0.6	-	0.7	-	-	-	-	-	-	-
Nocardioidaceae	0.6	-	-	-	-	0.6	0.6	-	0.7	-	-	-	-	-	-	-
Nocardioides	-	-	-	-	-	-	0.6	-		-	-	-	-	-	-	-
Aeromicrobium	0.6	-	-	-	-	-	-	-	0.7	-	-	-	-	-	-	-
Unclassified Nocarioidaceae	-	-	-	-	-	0.6	-	-	-	-	-	-	-	-	-	-
Probionibacteriaceae	-	-	0.6	-	-	-	-	-	-	-	-	-	-	-	-	-
Propionibacterium	-	-	0.6	-	-	-	-	-	-	-	-	-	-	-	-	-
Micrococcineae	1.9	4.2	1.7	2.5	2.9	2.9	1.8	4.0	0.7	-	-	3.1	-	1.1	2.5	2.1
Microbacteriaceae	-	1.2	-	0.6	0.6	1.1	-	2.3	-	-	-	2.1	-	-	-	-
Microbacterium	-	-	-	0.6	-	-	-	1.1	-	-	-	1.0	-	-	-	-
Leifsonia	-	1.2	-	-	0.6	0.6	-	-	-	-	-	1.0	-	-	-	-
Plantibacter	-	-	-	-	-	-	-	1.1	-	-	-		-	-	-	-
Unclassified Microbacteriaceae	-	-	-	-	-	0.6	-	-	-	-	-	-	-	-	-	-
Micrococcaceae	1.9	3.0	1.7	1.9	2.4	1.7	1.8	1.7	0.7	-	-	1.0	-	1.1	2.5	2.1
Arthrobacter	1.9	3.0	1.7	1.9	2.4	1.7	1.8	1.7	0.7	-	-	1.0	-	1.1	1.2	2.1
Rhothia	-	-	-	-	-	-	-	-	-	-	-	-	-	-	1.2	-
Streptosporangineae	-	-	-	-	-	0.6	-	-	-	-	-	1.0	-	-	-	-
Thermomonosporaceae	-	-	-	-	-	0.6	-	-	-	-	-	1.0	-	-	-	-
Acntinocorallia	-	-	-	-	-	-	-	-	-	-	-	1.0	-	-	-	-
Unclassified Thermomonosporaceae	-	-	-	-	-	0.6	-	-	-	-	-	-	-	-	-	-
Cautenulisporineae	-	-	-	-	1.2	-	-	1.1	-	-	-	-	-	-	-	-
Cautenulisporaceae	-	-	-	-	1.2	-	-	1.1	-	-	-	-	-	-	-	-
Cautenulispora	-	-	-	-	1.2	-	-	1.1	-	-	-	-	-	-	-	-
Micromonosporineae	-	-	-	-	-	-	-	0.6	-	-	-	-	-	-	-	-
Micromonosporaceae	-	-	-	-	-	-	-	0.6	-	-	-	-	-	-	-	-
Longispora	-	-	-	-	-	-	-	0.6	-	-	-	-	-	-	-	-
Unclassified Actinomycetales	0.6	-	-	-	-	-	-	-	-	-	-	-	-	-	-	-
Rubrobacteridae	-	-	-	-	-	-	-	0.6	-	-	-	-	-	-	-	-
Solirubrobacterales	-	-	-	-	-	-	-	0.6	-	-	-	-	-	-	-	-
Unclassified Solirubrobacterales	-	-	-	-	-	-	-	0.6	-	-	-	-	-	-	-	-
Acidimicrobidae	-	-	-	-	-	-	-	-	-	-	-	1.0	-	-	-	-
Acidimicrobiales	-	-	-	-	-	-	-	-	-	-	-	1.0	-	-	-	-
Acidimicrobineae	-	-	-	-	-	-	-	-	-	-	-	1.0	-	-	-	-
Unclassified Acidimicrobineae	-	-	-	-	-	-	-	-	-	-	-	1.0	-	-	-	-
Unclassfied Actinobacteria	-	-	0.6	-		-	-	-	-	-	-	-	-	-	-	-
Bacteroidetes	-	-	-	-	1.8	-	-	-	-	-	-	-	-	-	-	-
Flavobacteria	-	-	-	-	1.8	-	-	-	-	-	-	-	-	-	-	-
Flavobacteriales	-	-	-	-	1.8	-	-	-	-	-	-	-	-	-	-	-
Flavobacteriaceae	-	-	-	-	1.8	-	-	-	-	-	-	-	-	-	-	-
Flavobacterium	-	-	-	-	1.8	-	-	-	-	-	-	-	-	-	-	-
Chloroflexi	0.6	-	-	0.6	-	0.6	-	-	-	-	-	-	-	-	-	-
Ktedonobacteria	0.6	-	-	0.6	-		-	-	-	-	-	-	-	-	-	-
Ktedonobacterales	0.6	-	-	0.6	-		-	-	-	-	-	-	-	-	-	-
Ktedonobacteraceae	-	-	-	0.6	-		-	-	-	-	-	-	-	-	-	-
Ktedonobacter	-	-	-	0.6	-		-	-	-	-	-	-	-	-	-	-

Unclassified Ktedonobacterales	0.6	-	-	-	-		-	-	-	-	-	-	-	-	-	-
Thermomicrobia	-	-	-	-	-	0.6	-	-	-	-	-	-	-	-	-	-
Sphaerobacteridae	-	-	-	-	-	0.6	-	-	-	-	-	-	-	-	-	-
Sphaerobacteriales	-	-	-	-	-	0.6	-	-	-	-	-	-	-	-	-	-
Sphaerobacterineae	-	-	-	-	-	0.6	-	-	-	-	-	-	-	-	-	-
Sphaerobacteraceae	-	-	-	-	-	0.6	-	-	-	-	-	-	-	-	-	-
Sphaerobacter	-	-	-	-	-	0.6	-	-	-	-	-	-	-	-	-	-
Firmicutes	21.8	18.2	12.7*	19.8	13.5	10.3**	18.9	15.8	38.5	35.8	69.8**	59.8**	50.7	26.1	9.9**	41.1
Clostridia	-	-	-	0.6	0.6	-	-	-	0.7	-	7.0*	4.1	1.4	1.1	-	-
Clostridiales	-	-	-	-	-	-	-	-	0.7	-	7.0*	2.1	1.4	1.1	-	-
Peptococcaceae 1	-	-	-	-	-	-	-	-	0.7	-	2.3	1.0	-	-	-	-
Desulfosporosinus	-	-	-	-	-	-	-	-	0.7	-	2.3	1.0	-	-	-	-
Clostridiaceae 1	-	-	-	-	-	-	-	-	-	-	4.7*	-	-	1.1	-	-
Clostridium sensu strico	-	-	-	-	-	-	-	-	-	-	4.7*	-	-	1.1	-	-
Ruminococcaceae	-	-	-	-	-	-	-	-	-	-	-	-	1.4	-	-	-
Clostridium III	-	-	-	-	-	-	-	-	-	-	-	-	1.4	-	-	-
Unclassified Clostridia	-	-	-	0.6	0.6	-			-	-	-	2.1	-	-	-	-
Bacilli	21.8	18.2	12.7*	19.2	12.9*	10.3**	18.9	15.8	36.3	35.8	60.5**	54.6**	49.3	25.0	8.6**	37.9
Bacillales	21.8	18.2	12.7*	19.2	12.9*	10.3**	18.9	15.8	36.3	35.8	60.5**	54.6**	49.3	25.0	8.6**	37.9
Paenibacillaceae 1	18.6	12.1	8.1**	16.7	12.9	8.6**	12.2	10.7*	3.7	3.8	-	7.0	14.5**	4.3	-	4.2
Paenibacillus	18.6	12.1	8.1**	16.7	12.9	8.6**	12.2	10.2*	3.0	3.8	-	3.1	13.0**	4.3	-	3.2
Cohnella	-	-	-	-	-	-	-	-	0.7	-	-	3.1	-	-	-	-
Unclassified Paenibacillus 1	-	-	-	-	-	-	-	0.6	-	-	-	1.0	1.4	-	-	1.1
Bacillaceae 1	2.6	5.5	4.6	2.5	-	1.7	6.1	5.1	32.6	30.2	60.5**	41.2	30.4	19.6*	8.6**	31.6
Bacillus	2.6	5.5	4.6	2.5	-	1.7	5.5	5.1	32.6	30.2	58.1**	40.2	29.0	18.5*	8.6**	32.6
Falsibacillus	-	-	-	-	-	-	-	-	-	-	-	1.0	-	-	-	-
Unclassified Bacillaceae 1	-	-	-	-	-	-	0.6	-	-	-	2.3	-	1.4	-	-	1.1
Bacillales Incertae Sedis	-	0.6	-	-	-	-	-	-	-	-	-	-	-	-	-	-
Gemmela	-	0.6	-	-	-	-	-	-	-	-	-	-	-	-	-	-
Staphylococcaceae	-	-	-	-	-	-	0.6	-	-	-	-	2.1	2.9	-	-	-
Staphylococcus	-	-	-	-	-	-	0.6	-	-	-	-	2.1	2.9	-	-	-
Planococcaceae	-	-	-	-	-	-	-	-	-	1.9	-	2.1	-	1.1	-	1.1
Lysinibacillus	-	-	-	-	-	-	-	-	-	-	-	1.0	-	-	-	-
Ureibacillus	-	-	-	-	-	-	-	-	-	-	-	1.0	-	1.1	-	-
Sporosarcina	-	-	-	-	-	-	-	-	-	-	-	-	-	-	-	1.1
Planococcaceae incertae sedis	-	-	-	-	-	-	-	-	-	1.9	-	-	-	-	-	-
Unclassified Bacillales	0.6	-	-	-	-	-	-	-	-	-	-	2.1	1.4	1.1	-	-
Negativexutes	-	-	-	-	-	-	-	-	-	-	-	1.0	-	-	-	-
Selenomonadales	-	-	-	-	-	-	-	-	-	-	-	1.0	-	-	-	-
Veillonellaceae	-	-	-	-	-	-	-	-	-	-	-	1.0	-	-	-	-
Unclassified Veillonellaceae	-	-	-	-	-	-	-	-	-	-	-	1.0	-	-	-	-
Unclassified Firmicutes	-	-	-	-	-	-	-	-	1.5	-	2.3	-	-	-	-	2.1
Gemmatimonadetes	-	-	-	-	-	0.6	-	-	-	-	-	-	-	-	-	-
Gemmatimonadetes	-	-	-	-	-	0.6	-	-	-	-	-	-	-	-	-	-
Gemmatimonadales	-	-	-	-	-	0.6	-	-	-	-	-	-	-	-	-	-
Gemmatimonadaceae	-	-	-	-	-	0.6	-	-	-	-	-	-	-	-	-	-
Gemmatimonas	-	-	-	-	-	0.6	-	-	-	-	-	-	-	-	-	-
Planctomycetes	5.1	2.4	3.5	2.5	4.7	9.7	4.9	1.7	-	-	-	1.0	-	3.3	2.5	-
Planctomycetacia	5.1	2.4	3.5	2.5	4.7	9.7	4.9	1.7	-	-	-	1.0	-	3.3	2.5	-
Planctomycetales	5.1	2.4	3.5	2.5	4.7	9.7	4.9	1.7	-	-	-	1.0	-	3.3	2.5	-
Planctomycetaceae	5.1	2.4	3.5	2.5	4.7	9.7	4.9	1.7	-	-	-	1.0	-	3.3	2.5	-
Planctomyces	0.6	0.6	0.6	0.6	1.8	0.6	-	-	-	-	-	-	-	-	-	-
Schlesneria	1.3	1.2	0.6	-	-	4.0	1.8	0.6	-	-	-	-	-	-	-	-
Zavarzinella	1.9	0.6	0.6	0.6	0.6	1.7	0.6	0.6	-	-	-	-	-	2.2	2.5	-
Singulisphaera	-	-	1.2	0.6	1.8	0.6	1.2	0.6	-	-	-	1.0	-	1.1	-	-
Gemmata	-	-	-	-	-	1.1	-	-	-	-	-	-	-	-	-	-
Unclassified Planctomycetaceae	1.3	-	0.6	0.6	0.6	1.7	1.2	0.6	-	-	-	-	-	-	-	-
Proteobacteria	57.7	67.3	72.8**	73.5**	64.7	62.9	64.0	68.4*	57.8	60.4	23.3**	27.8**	40.6	51.1	74.1*	35.8**
Alphaproteobacteria	13.5	21.2	26.6**	27.2**	28.2**	36.6**	18.9	32.8**	49.6	45.3	18.6**	19.6**	31.9	43.5	65.4*	18.9**
Rhizobiales	12.8	17.6	26.6**	21.9*	23.5*	36.0**	18.9	31.1**	48.1	20.8**	16.3**	18.6	24.6**	43.5	65.4*	15.8**
Phyllobacteriaceae	0.6	6.1**	5.2*	1.2	1.2	3.4	2.4	1.7	44.4	13.2**	7.0**	10.3	7.2**	31.5	50.6	1.1**
Phyllobacterium	-	2.4	-	-	-	-	0.6	-	44.4	13.2**	7.0**	7.2**	7.2**	30.4*	48.1	1.1**
Meshorizobium	-	3.0*	4.0*	0.6	1.2	2.9*	1.2	1.7	-	-	-	3.1	-	1.1	1.2	-
Unclassified Phyllobacteriaceae	0.6	0.6	1.2	0.6	-	0.6	0.6	-	-	-	-	-	-	-	1.2	-
Bradyrhizobiaceae	5.8	3.0	4.6	1.9	7.6	3.4	5.5	6.2	0.7	3.8	-	2.1	4.3	3.3	1.2	5.3*
Bosea	0.6	0.6	0.6	-	0.6	0.6	-	1.1	-	1.9	-		1.4	1.1	-	3.2
Bradyrhizobium	-	0.6	1.2	0.6	0.6	-	2.4	0.6	0.7	-	-	1.0	2.9	-	-	-
Afipia	-	-	-	-	0.6	-	-	-	-	-	-	-	-	-	-	-
Unclassified Bradyrhizobiaceae	5.1	1.8	2.9	1.2	5.9	2.9	3.0	4.5	-	1.9	-	1.0	-	2.2	1.2	2.1

Rhizobiaceae	3.2	6.7	13.3**	15.4**	8.8*	22.3**	8.5	16.9**	2.2	1.9	4.7	-	4.3	8.7*	9.9*	5.3
Rhizobium	2.6	6.7	13.3**	14.8**	8.2*	21.1**	7.9*	16.4**	2.2	1.9	4.7	-	4.3	8.7*	9.9*	5.3
Kaistia	0.6	-	-	0.6	0.6	1.1	0.6	0.6	-	-	-	-	-	-	-	-
Methylobacteriaceae	-	-	-	-	0.6	-	-	-	-	-	2.3	6.2**	7.2**	-	2.5	3.2
Methylobacterium	-	-	-	-	0.6	-	-	-	-	-	2.3	6.2**	7.2**	-	2.5	3.2
Hyphomicrobiaceae	2.6	1.2	1.7	1.9	2.4	1.1	1.2	2.3	-	1.9	-	-	1.4	-	1.2	-
Devosia	1.9	1.2	1.7	1.9	2.4	1.1	1.2	2.3	-	-	-	-	1.4	-	1.2	-
Hyphomicrobium	0.6	-	-	-	-	-	-	- -	-	1.9	-	-		-	-	-
Brucellaceae	0.6	-	-	1.2	1.2	3.4	-	2.3	-	-	-	-	-	-	-	-
Ochrobactrum	0.6	-	-	1.2	1.2	3.4	-	2.3	-	-	-	-	-	-	-	-
Xanthobacteraceae	-	0.6	0.6	-	0.6	1.1	0.6	0.6	-	-	-	-	-	-	-	1.1
Pseudolabrys	-	0.6	0.6	-			0.6	0.6	-	-	-	-	-	-	-	
Labrys	-	-	-	-	0.6	1.1	-	-	-	-	-	-	-	-	-	1.1
Methylocystaceae	-	-	-	-	-	0.6	-	-	-	-	-	-	-	-	-	-
Methylocystis	-	-	-	-	-	0.6	-	-	-	-	-	-	-	-	-	-
Aurantimonadaceae	-	-	-	-	-	-	-	-	-	-	2.3	-	-	-	-	-
Aurantimonas	-	-	-	-	-	-	-	-	-	-	2.3	-	-	-	-	-
Unclassified Rhizobiales	-	-	1.2	-	1.2	0.6	0.6	1.1	0.7	-	-	-	-	-	-	-
Sphingomonadales	0.6	3.0	-	4.9*	2.4	0.6	-	0.1	0.7	22.6**	2.3	-	7.2	-	-	2.1
Sphingomonadaceae	0.6	3.0	-	4.9*	2.4	0.6	-	-	0.7	22.6**	2.3	-	7.2	-	-	2.1
Novosphingobium	-	0.6	-		0.6	-	-	-	-	-	-	-	-	-	-	-
Sphingomonas	-		-	3.7*	0.6	0.6	-	-	0.7	22.6**	2.3	-	7.2	-	-	2.1
Sphingobium	-	1.2	-	0.6	-	-	-	-	-	-	-	-		-	-	-
Unclassified Sphingomonadaceae	0.6	1.2	-	0.6	1.2	-	-	-	-	-	-	-	-	-	-	-
Rhodospirillales	-	-	-	-	-	-	-	-	0.7	1.9	-	0.1	-	-	-	-
Acetobacteraceae	-	-	-	-	-	-	-	-	0.7	1.9	-	0.1	-	-	-	-
Roseomonas	-	-	-	-	-	-	-	-	0.7	1.9	-		-	-	-	-
Acidisphaera	-	-	-	-	-	-	-	-			-	0.1	-	-	-	-
Unclassified Alphaproteobacteria	-	0.6	-	0.6	2.9	-	-	0.6			-		-	-	-	1.1
Betaproteobacteria	3.2	10.3*	3.5	7.4	5.9	5.7	8.5	5.6	1.5	5.7	-	1.0	-	4.3	4.9	1.1
Burkholderiales	3.2	9.7*	3.5	7.4	5.9*	5.7	8.5	4.5	1.5	5.7	-	1.0	-	4.3	4.9	1.1
Comamonadaceae	1.3	4.8	-	4.9	2.9	5.1	4.3	1.7	0.7	1.9	-	1.0	-	4.3	4.9	1.1
Variovorax	-	-	-	-	0.6	-	1.2	-	-	-	-	-	-	-	2.5	1.1
Roseateles	-	-	-	-	-	-	-	-	-	-	-	-	-	-	-	-
Polaromonas	0.6	3.0	-	1.9	0.6	4.0	2.4	1.1	-	1.9	-	-	-	3.3	2.5	-
Comamonas	-	1.8	-	3.1*	1.8	0.6	0.6	0.6	-	-	-	1.0	-	-	-	-
Delftia	0.6	-	-	-	-	-	-	-	-	-	-	-	-	-	-	-
Acidovorax	-	-	-	-	-	-	-	-	0.7	-	-	-	-	-	-	-
Unclassified Comamonadaceae	-	-	-	-	-	0.6	-	-	-	-	-	-	-	1.1	-	-
Oxalobacteraceae	1.3	1.2	0.6	1.2	-	-	1.2	1.1	0.7	3.8	-	-	-	-	-	-
Collimonas	-	1.2	-		-	-	-	-	-	-	-	-	-	-	-	-
Herbaspirillum	1.3	-	-	1.2	-	-	-	0.6	0.7	-	-	-	-	-	-	-
Unclassified Oxalobacteraceae	-	-	0.6	-	-	-	1.2	0.6	-	3.8	-	-	-	-	-	-
Alcaligenaceae	0.6	2.4	1.7	-	-	-	1.2	1.1	-	-	-	-	-	-	-	-
Pusillimonas	-	1.2	-	-	-	-	0.6	-	-	-	-	-	-	-	-	-
Achromobacter	-	-	0.6	-	-	-	0.6	-	-	-	-	-	-	-	-	-
Unclassified Alcaligenaceae	0.6	1.2	1.2	-	-	-	0.0	1.1	-	-	-	-	-	-	-	-
Burkholderiaceae	-	1.2	1.2	1.2	2.9	0.6	1.2	0.6	-	-	-	-	-	-	-	-
Pandoraea	-	0.6	-	-	-	-	0.6	-	-	-	-	-	-	-	-	-
Burkholderia	-	0.6	1.2	1.2	1.8	0.6	0.6	0.6	-	-	-	-	-	-	-	-
Ralstonia	-	-	-	-	0.6	-	-	-	-	-	-	-	-	-	-	-
Cupriavidus	-	-	-	-	0.6	-	-	-	-	-	-	-	-	-	-	-
Unclassified Burkholderiales	-	-	-	-	-	-	0.6	-	-	-	-	-	-	-	-	-
Shinella	-	-	-	-	-	-	-	0.6	-	-	-	-	-	-	-	-
Unclassified Betaproteobacteria	-	0.6	-	-	-	-	-	0.6	-	-	-	-	-	-	-	-
Gammaproteobacteria	33.3	35.2	37.0	37.0	28.2	19.4**	27.4	24.3	5.9	7.6	4.7	6.2	7.2	3.3	2.5	15.8*
Legionellales	18.6	16.4	10.4*	13.0	7.6**	10.9*	12.2*	7.3**	0.7	1.9	-	3.1	-	1.1	-	-
Coxiellaceae	18.6	15.8	9.2*	13.0	7.1**	9.1*	8.5**	6.8**	-	1.9	-	3.1	-	1.1	-	-
Aquicella	18.6	15.8	9.2*	13.0	7.1**	9.1*	7.9**	6.8**	-	1.9	-	3.1	-	1.1	-	-
Coxiella	-	-	-	-	-	-	0.6	-	-	-	-	-	-	-	-	-
Legionellaceae	-	-	1.2	-	0.6	1.7	3.7*	0.6	0.7	-	-	-	-	-	-	-
Legionella	-	-	1.2	-	0.6	1.7	3.7	0.6	0.7	-	-	-	-	-	-	-
Enterobacteriales	-	2.4	4.6**	8.6**	5.3**	1.1	1.2	5.1**	-	-	-	1.0	-	-	-	-
Enterobacteriaceae	-	2.4	4.6**	8.6**	5.3**	1.1	1.2	5.1**	-	-	-	1.0	-	-	-	-
Serratia	-	0.6	-	-	0.6	-	-	-	-	-	-	-	-	-	-	-
Enterobacter	-	1.2	1.2	1.2	-	-	-	0.6	-	-	-	-	-	-	-	-
Citrobacter	-	-	1.7	-	-	-	-	-	-	-	-	-	-	-	-	-
Pantoea	-	-	0.6	3.1*	1.2	-	0.6	-	-	-	-	-	-	-	-	-
Erwinia	-	-	-	-	1.8	-	-	-	-	-	-	-	-	-	-	-

Pectobacterium	-	-	-	-	1.2	0.6	-	2.3	-	-	-	-	-	-	-	-
Yersinia	-	-	-	-	-	-	0.6	-	-	-	-	-	-	-	-	-
Unclassified Enterobateriaceae	-	0.6	1.2	4.3	0.6	0.6	0.0	2.3			-	1.0	-	-	-	-
Pseudomonadales	-	2.4	3.5*	4.9**	6.5**	1.7	1.2	3.4*	0.7	5.7*	4.7	-	4.3	-	1.2	14.7**
Moraxellaceae	-	-	-	2.5	5.3**	0.6	-	0.6	-	-	-	-	-	-	-	10.5**
Acinetobacter	-	-	-	2.5	5.3**	0.6	-	0.6	-	-	-	-	-	-	-	-
Enhydrobacter	-	-	-	-	-	-	-	-	-	-	-	-	-	-	-	10.5**
Pseudomonadaceae	-	2.4	3.5*	2.5	1.2	1.1	1.2	2.8*	0.7	5.7*	4.7	-	4.3	-	1.2	4.2
Pseudomonas	-	2.4	3.5*	2.5	1.2	1.1	1.2	2.8*	0.7	5.7*	4.7	-	4.3	-	1.2	4.2
Rhizobacter	-	-	-	-	-	-	-	-	-	-	-	-	-	-	-	-
Xantomonadales	1.3	4.2	0.6	1.2	4.7	0.6	1.2	2.8	4.4	-	-	1.0	1.4	1.1	-	-*
Xantomonadaceae	1.3	4.2	0.6	1.2	4.7	0.6	1.2	2.8	4.4	-	-	1.0	1.4	1.1	-	-*
Rudaea	0.6	1.2	-	-	-	0.6	1.2	-	-	-	-	-	-	-	-	-
Stenotrophomonas	-	-	0.6	-	0.6	-	-	0.6	4.4	-	-	-	1.4	-	-	-*
Dokdonella	-	-	-	-	-	-	-	0.6	-	-	-	-	-	-	-	-
Rhodanobacter	0.6	1.8	-	1.2	1.8	-	-	1.1	-	-	-	-	-	-	-	-
Lysobacter	-	1.2	-	-	1.8	-	-	-	-	-	-	1.0	-	1.1	-	-
Luteibacter	-	-	-	-	-	-	-	0.6	-	-	-	-	-	-	-	-
Unclassified Xanthomonadaceae	-	-	-	-	0.6	-	-	-	-	-	-	-	-	-	-	-
Thiotrichales	0.6	-	0.6	-		-	0.6	0.6	-	-	-	-	-	-	-	-
Thiotrichales incertae sedis	-	-	-	-	-	-	0.6	-	-	-	-	-	-	-	-	-
Unclassified thiotrichales	0.6	-	0.6	-	-	-	-	0.6	-	-	-	-	-	-	-	-
Chromatiales	-	-	0.6	-	-	-	-	-	-	-	-	-	-	-	-	-
Unclassified Chromatiales	-	-	0.6	-	-	-	-	-	-	-	-	-	-	-	-	-
Unclassified Gammaproteobacteria	12.8	9.7	16.8	9.3	4.1	5.1	11.0	5.1	-	-	-	1.0	1.4	1.1	1.2	1.1
Deltaproteobacteria	6.4	0.6**	5.8	1.2*	1.2*	1.1*	7.3	5.6	-	1.9	-	1.0	-	-	-	-
Myxococcales	5.8	0.6**	5.8	1.2*	1.2*	1.1*	6.7	5.6	-	-	-	-	-	-	-	-
Cystobacterineae	5.1	0.6*	5.8	1.2	1.2*	1.1*	6.7	5.6	-	-	-	-	-	-	-	-
Unclasified Cystobacterineae	5.1	0.6	1.2	-	1.2	1.1	6.7	5.6	-	-	-	-	-	-	-	-
Sorangiineae	0.6	-	-	-	-	-	-	-	-	-	-	-	-	-	-	-
Unclassified Sorangiineae	0.6	-	-	-	-	-	-	-	-	-	-	-	-	-	-	-
Bdellovibrionales	-	-	-	-	-	-	0.6	-	-	-	-	-	-	-	-	-
Bdellovibrionaceae	-	-	-	-	-	-	0.6	-	-	-	-	-	-	-	-	-
Bdellovibrio	-	-	-	-	-	-	0.6	-	-	-	-	-	-	-	-	-
Unclassfied Deltaproteobacteria	0.6	-	-	-	-	-	-	-	-	1.9	-	1.0	-	-	-	-
Unclassified Proteobacteria	1.3	-	-	0.6	0.6	0.0	1.8	-	0.7	-	-	-	1.4	-	1.2	-
Verrucomicrobia	1.3	1.8	1.2	0.0	3.5	5.7*	3.7	4.0	-	-	-	-	-	2.2	-	-
Spartobacteria	0.6	1.2	-	-	1.8	4.6*	1.8	2.3	-	-	-	-	-	2.2	-	-
Spartobacteria genera incertae sedis	0.6	1.2	-	-	1.8	4.6*	1.8	2.3	-	-	-	-	-	2.2	-	-
Opitutae	0.6	0.6	1.2	-	-	0.6	1.2	1.1	-	-	-	-	-	-	-	-
Puniceicoccales	0.6	0.6	1.2	-	-	0.6	1.2	1.1	-	-	-	-	-	-	-	-
Puniceicoccaceae	0.6	0.6	1.2	-	-	0.6	1.2	1.1	-	-	-	-	-	-	-	-
Unclassified Puniceicoccaceae	0.6	0.6	1.2	-	-	0.6	1.2	1.1	-	-	-	-	-	-	-	-
Verrucomicrobiae	-	-	-	-	1.8	0.6	-	-	-	-	-	-	-	-	-	-
Verrucomicrobiales	-	-	-	-	1.8	0.6	-	-	-	-	-	-	-	-	-	-
Verrucomicrobiaceae	-	-	-	-	1.8	0.6	-	-	-	-	-	-	-	-	-	-
Luteolibacter	-	6.0	-	-	-	0.6	-	-	-	-	-	-	-	-	-	-
Verrucomicrobium	0.6	-	-	-	0.6	-	-	-	-	-	-	-	-	-	-	-
Prosthecobacter	1.2	-	-	-	1.2	-	-	-	-	-	-	-	-	-	-	-
Unclassfied Verrucomicrobia	-	-	-	-	-	-	0.6	0.6	-	-	-	-	-	-	-	-
Bacteria_incertae_sedis	-	-	-	0.6	1.8	0.6	-	-	-	-	-	-	-	-	-	-
Unclassified Bacteria	1.9	1.2	1.2	-	-	1.7	2.4	0.6	0.7	-	4.7	1.0	-	2.2	-	-

[a]Sequences were grouped using the RDP Classifier of the Ribosomal Database Project-II release 11 with a confidence threshold of 80%.

[b]R, M and S stand for phenotypes of potato 8 genotypes which are resistant, medium resistant and susceptible to potato scab diseases, respectively. R1, R2, R3, M1, M2, S1, S2 and S3 are designated for clone libraries of 8 potato genotypes (Yukirasha, 02005-10, Snow March, Star Rubi, Snowden, Irish Cobbler, Toyoshiro, and Piruka, respectively). CD stands for the isolate collections derived

* and ** indicate statistical significance at the 5%and 1% levels ($P < 0.05$ and $P < 0.01$), respectively, based on the results of Fisher's exact test between R1 and other libraries.

Supplementary Table 2. Relative abundance of clones in each library of 16S rRNA gene sequences for root- and tuber-associated bacteria of 8 potato genotypes

	Reltative abundnce in clone libraries (%)																		
	Root								Tuber									Identity	
OTUs	R1[a]	R2	R3	M1	M2	S1	S2	S3	R1	R2	R3	M1	M2	S1	S2	S3	Closest known species	(%)	Accession No.
AP1	-	-	-	-	-	-	-	-	-	-	2.3	6.2	-	-	1.2	1.1	*Methylobacterium mesophilicum*	100	AB900968
AP2	-	-	-	-	-	-	-	-	-	-	-	-	-	-	1.2	-	*Methylobacterium hispanicum*	99	KF371658
AP3	-	-	-	-	0.6	-	-	-	-	-	-	-	-	-	-	-	*Methylobacterium adhaesivum*	99	AB698720
AP4	-	-	-	-	-	-	-	-	-	-	-	-	7.2	-	-	-	*Methylobacterium thiocyanatum*	100	LK020748
AP5	-	-	-	-	-	-	-	-	-	-	-	-	-	-	-	2.1	*Methylobacterium platani*	99	JX311414
AP6	0.6	0.6	0.6	-	0.6	0.6	-	1.1	-	-	-	-	1.4	1.1	-	3.2	*Bosea thiooxidans*	100	HG794268
AP7	-	-	-	-	-	-	-	-	-	1.9	-	-	-	-	-	-	*Bosea lupini*	95	KF730777
AP8	4.5	0.6	2.3	1.2	4.7	-	3.0	1.7	-	-	-	1.0	2.9	-	1.2	2.1	*Bradyrhizobium elkanii*	99	FN178446
AP9	-	-	-	0.6	-	-	-	-	-	-	-	-	-	1.1	-	-	*Afipia broomeae*	97	HF558416
AP10	0.6	1.2	1.7	-	2.4	2.9	2.4	3.4	0.7	1.9	-	1.0	-	1.1	-	-	*Tardiphaga robiniae*	100	NR_117178
AP11	-	-	0.6	-	-	-	-	-	-	-	-	-	-	-	-	-	*Pseudolabrys taiwanensis*	96	EU938323
AP12	-	-	-	-	-	-	0.6	-	-	-	-	-	-	-	-	-	*Pseudolabrys taiwanensis*	98	EU938323
AP13	-	0.6	0.6	-	-	-	-	0.6	-	-	-	-	-	-	-	-	*Pseudolabrys taiwanensis*	95	EU938323
AP14	-	-	-	-	0.6	-	-	-	-	-	-	-	-	-	-	-	*Labrys methylaminiphilus*	98	NR_041336
AP15	-	-	-	-	-	1.1	-	-	-	-	-	-	-	-	-	1.1	*Labrys wisconsinensis*	99	FJ707292
AP16	-	-	-	-	-	0.6	-	-	-	-	-	-	-	-	-	-	*Alsobacter metallidurans*	96	AB231946
AP17	-	-	-	-	-	-	-	-	0.7	-	-	-	-	-	-	-	*Methylocystis bryophila*	95	NR_108474
AP18	1.3	-	1.2	-	1.2	-	0.6	1.7	-	-	-	-	1.4	-	-	-	*Devosia psychrophila*	99	NR_117516
AP19	-	-	-	0.6	1.2	1.1	-	0.6	-	-	-	-	-	-	1.2	-	*Devosia insulae*	99	NR_044036
AP20	0.6	1.2	0.6	1.2	-	-	0.6	0.6	-	-	-	-	-	-	-	-	*Devosia chinhatensis*	96	JX971555
AP21	-	-	-	-	-	-	0.6	-	-	-	-	-	-	-	-	-	*Nordella oligomobilis*	92	NR_114615
AP22	-	-	-	-	-	-	-	0.6	-	-	-	-	-	-	-	-	*Nordella oligomobilis*	97	NR_114615
AP23	0.6	-	-	-	-	-	-	-	-	1.9	-	-	-	-	-	-	*Hyphomicrobium facile*	100	Y14312
AP24	0.6	-	-	0.6	0.6	1.1	0.6	0.6	-	-	-	-	-	-	-	-	*Kaistia granuli*	100	KF981585
AP25	-	-	-	1.9	1.8	4.6	-	1.1	-	-	-	-	-	1.1	-	-	*Rhizobium larrymoorei*	100	KJ472824
AP26	-	-	-	1.2	-	0.6	1.2	3.4	-	-	-	-	-	1.1	-	1.1	*Rhizobium radiobacter*	100	KF673154
AP27	0.6	2.4	9.8	5.6	4.1	11.4	1.8	8.5	-	-	2.3	-	2.9	5.4	3.7	3.2	*Rhizobium radiobacter*	100	KJ719245
AP28	-	-	-	-	-	0.6	-	0.6	-	-	-	-	-	-	1.2	-	*Rhizobium daejeonense*	99	HF585487
AP29	0.6	-	-	-	-	0.6	2.4	-	-	-	-	-	-	-	-	-	*Rhizobium herbae*	99	JX841043
AP30	-	-	-	-	-	-	-	-	0.7	-	-	-	-	-	-	-	*Rhizobium tubonense*	100	HM070185
AP31	1.9	4.2	2.9	4.3	2.4	2.9	3.7	2.3	1.5	1.9	-	-	1.4	1.1	4.9	1.1	*Rhizobium etli*	99	AM921623
AP32	-	-	0.6	2.5	-	0.6	-	1.1	0.7	-	2.3	-	-	-	-	-	*Rhizobium alamii*	100	GU552885
AP33	0.6	-	-	0.6	1.2	2.3	-	2.3	-	-	-	-	-	-	-	-	*Ochrobactrum pseudogrignonense*	99	KF844052
AP34	-	-	-	0.6	-	1.1	-	-	-	-	-	-	-	-	-	-	*Ochrobactrum anthropi*	99	KJ415176
AP35	-	0.6	0.6	0.6	-	-	-	-	-	-	-	-	-	-	-	-	*Mesorhizobium tianshanense*	97	JF730145
AP36	-	-	1.2	-	-	-	-	-	-	-	-	-	-	-	-	-	*Mesorhizobium thiogangeticum*	98	KF876855
AP37	-	3.0	3.5	0.6	1.2	2.9	1.2	1.7	-	-	-	3.1	-	1.1	1.2	-	*Mesorhizobium loti*	100	KJ529052
AP38	-	-	0.6	-	-	0.6	0.6	-	-	-	-	-	-	-	1.2	-	*Aminobacter anthyllidis*	98	HG974525
AP39	-	2.4	-	-	-	-	0.6	-	43.7	13.2	7.0	7.2	7.2	30.4	48.1	1.1	*Phyllobacterium myrsinacearum*	100	KJ147080
AP40	-	-	-	-	-	-	-	-	0.7	-	-	-	-	-	-	-	*Phyllobacterium myrsinacearum*	97	KJ147080
AP41	0.6	-	-	-	-	-	-	-	-	-	-	-	-	-	-	-	*Aquamicrobium defluvii*	98	KC488623
AP42	-	-	-	-	-	0.6	-	-	-	-	-	-	-	-	-	-	*Mesorhizobium thiogangeticum*	98	AB675379
AP43	-	-	-	-	-	-	-	-	-	-	2.3	-	-	-	-	-	*Aureimonas ureilytica*	99	AB682441
AP44	-	-	-	-	0.6	-	-	-	-	-	-	-	-	-	-	-	*Rhizobium oryzae*	96	NR_044393
AP45	-	-	-	-	0.6	-	-	-	-	-	-	-	-	-	-	-	*Blastobacter capsulatus*	94	NR_026116
AP46	-	-	-	-	0.6	-	-	-	-	-	-	-	-	-	-	-	*Rhodovulum euryhalinum*	88	NR_043406
AP47	-	0.6	-	-	1.2	-	-	-	-	-	-	-	-	-	-	1.1	*Enhydrobacter aerosaccus*	97	AB641400
AP48	-	-	-	-	0.6	-	-	0.6	-	-	-	-	-	-	-	-	*Reyranella massiliensis*	99	HM048834
AP49	-	-	-	-	0.6	-	-	-	-	-	-	-	-	-	-	-	*Rhodoligotrophos appendicifer*	90	NR_113322
AP50	-	0.6	-	-	-	-	-	-	-	-	-	-	-	-	-	-	*Afipia massiliensis*	94	NR_025646
AP51	-	-	-	0.6	-	-	-	-	-	-	-	-	-	-	-	-	*Sphingomonas melonis*	99	JF459931
AP52	-	-	-	0.6	0.6	-	-	-	-	-	-	-	-	-	-	-	*Sphingomonas aerolata*	100	HG934368
AP53	-	-	-	-	-	-	-	0.6	-	-	-	-	-	-	-	-	*Sphingomonas mali*	100	NR_113762
AP54	-	-	-	-	-	-	-	-	-	1.9	-	-	-	-	-	-	*Sphingomonas phyllosphaerae*	99	JQ659446
AP55	-	-	-	0.6	-	-	-	-	0.7	20.8	2.3	-	7.2	-	-	2.1	*Sphingomonas paucimobilis*	100	KJ472818
AP56	-	-	-	1.2	-	-	-	-	-	-	-	-	-	-	-	-	*Sphingomonas humi*	98	JX094162
AP57	-	0.6	-	1.2	-	0.6	-	-	-	-	-	-	-	-	-	-	*Sphingomonas jaspsi*	99	KC921161
AP58	0.6	0.6	-	-	1.2	-	-	-	-	-	-	-	-	-	-	-	*Sphingomonas jaspsi*	97	KF580874
AP59	-	1.2	-	0.6	-	-	-	0.6	-	-	-	-	-	-	-	-	*Sphingobium cloacae*	99	NR_114135
AP60	-	0.6	-	-	0.6	-	-	-	-	-	-	-	-	-	-	-	*Novosphingobium lindaniclasticum*	99	NR_118312
AP61	1.3	-	0.6	-	-	-	-	-	-	-	-	-	-	-	-	-	*Candidatus* Nucleicultrix amoebiphila	88	KF697195
AP62	-	-	-	-	-	-	0.6	0.6	-	-	-	-	-	-	-	-	*Holospora obtusa*	88	X58198
AP63	-	-	-	-	0.6	-	-	-	-	-	-	-	-	-	-	-	*Candidatus* Nucleicultrix amoebiphila	94	KF697195
AP64	-	-	-	-	-	-	-	-	0.7	1.9	-	-	-	-	-	-	*Roseomonas mucosa*	99	JF938918
AP65	-	-	-	-	-	-	-	-	-	-	-	1.0	-	-	-	-	*Acidisphaera rubrifaciens*	95	NR_037119
BP1	-	-	-	2.5	-	-	0.6	-	-	-	-	-	-	-	-	-	*Comamonas testosteroni*	99	KJ806514
BP2	-	-	-	-	-	-	-	0.6	-	-	-	-	-	-	-	-	*Comamonas odontotermitis*	99	NR_043859
BP3	-	1.8	-	0.6	1.8	-	-	-	-	-	-	1.0	-	-	-	-	*Comamonas koreensis*	100	KF475801
BP4	0.6	-	-	-	-	-	-	-	-	-	-	-	-	-	-	-	*Delftia acidovorans*	99	DQ530071
BP5	-	-	-	-	-	-	-	-	-	-	-	-	-	1.1	-	-	*Delftia tsuruhatensis*	97	KJ147081
BP6	-	-	-	-	-	0.6	-	-	-	-	-	-	-	-	-	-	*Comamonas jiangduensis*	99	KJ726553
BP7	-	-	-	-	-	0.6	-	-	-	-	-	-	-	-	-	-	*Comamonas jiangduensis*	93	KJ726553
BP8	0.6	3.0	-	1.9	0.6	4.0	2.4	1.1	-	1.9	-	-	-	3.3	2.5	-	*Polaromonas rhizosphaerae*	99	EF127651
BP9	-	-	-	-	0.6	-	1.2	-	-	-	-	-	-	-	2.5	1.1	*Variovorax boronicumulans*	100	KJ472790
BP10	-	-	-	-	-	-	-	-	0.7	-	-	-	-	-	-	-	*Acidovorax defluvii*	98	KJ643429
BP11	-	-	-	-	-	-	1.2	-	-	-	-	-	-	-	-	-	*Herminiimonas fonticola*	97	AB512142
BP12	1.3	-	-	-	-	-	-	1.1	0.7	1.9	-	-	-	-	-	-	*Herbaspirillum seropedicae*	97	NR_114142
BP13	-	1.2	-	-	-	-	-	-	-	-	-	-	-	-	-	-	*Collimonas pratensis*	99	AY281143
BP14	-	-	-	1.2	-	-	-	-	-	-	-	-	-	-	-	-	*Herbaspirillum seropedicae*	98	HQ219858
BP15	-	-	-	-	0.6	-	-	-	-	-	-	-	-	-	-	-	*Ralstonia pickettii*	100	KJ806364
BP16	-	-	-	-	0.6	-	-	-	-	-	-	-	-	-	-	-	*Cupriavidus pampae*	100	NR_116994
BP17	-	-	-	-	-	-	0.6	-	-	1.9	-	-	-	-	-	-	*Herbaspirillum seropedicae*	96	HQ406764
BP18	-	1.2	-	-	-	-	0.6	-	-	-	-	-	-	-	-	-	*Pusillimonas terrae*	98	JF303042
BP19	0.6	1.2	1.2	-	-	-	-	1.1	-	-	-	-	-	-	-	-	*Paralcaligenes ureilyticus*	100	NR_116812
BP20	-	-	0.6	-	-	-	0.6	-	-	-	-	-	-	-	-	-	*Achromobacter spanius*	100	KJ726578
BP21	-	-	0.6	-	-	-	-	-	-	-	-	-	-	-	-	-	*Collimonas fungivorans*	92	AB740926
BP22	-	-	0.6	-	-	-	-	-	-	-	-	-	-	-	-	-	*Burkholderia cepacia*	100	KJ634149
BP23	-	0.6	0.6	1.2	1.8	0.6	0.6	0.6	-	-	-	-	-	-	-	-	*Burkholderia phytofirmans*	99	KF981504
BP24	-	0.6	-	-	-	-	0.6	-	-	-	-	-	-	-	-	-	*Pandoraea norimbergensis*	99	JX995138

BP25	-	0.6	-	-	-	-	-	-	-	-	-	-	-	-	-	-	*Burkholderia xenovorans*	92	EU723243
BP26	-	-	-	-	-	-	-	0.6	-	-	-	-	-	-	-	-	*Cupriavidus gilardii*	89	AY860231
GP1	0.6	-	-	0.6	-	-	-	-	-	-	-	-	-	-	-	-	*Legionella hackeliae*	87	NR_104894
GP2	-	-	-	-	-	-	-	0.6	-	-	-	-	-	-	-	-	*Legionella hackeliae*	89	NR_104894
GP3	-	-	-	-	-	-	-	0.6	-	-	-	-	-	-	-	-	*Legionella hackeliae*	88	NR_104894
GP4	-	-	-	-	-	-	0.6	-	-	-	-	-	-	-	-	-	*Legionella yabuuchiae*	86	AB233211
GP5	-	-	-	-	-	-	0.6	-	-	-	-	-	-	-	-	-	*Legionella cardiaca*	88	NR_109426
GP6	-	-	-	-	-	-	0.6	-	-	-	-	-	-	-	-	-	*Legionella hackeliae*	85	NR_104894
GP7	-	-	-	-	-	-	0.6	-	-	-	-	-	-	-	-	-	*Legionella hackeliae*	88	NR_104894
GP8	-	-	0.6	-	-	-	-	-	-	-	-	-	-	-	-	-	*Legionella cardiaca*	88	NR_109426
GP9	-	-	1.2	-	-	-	-	-	-	-	-	-	-	-	-	-	*Legionella londiniensis*	85	NR_044963
GP10	-	-	-	-	0.6	-	-	-	-	-	-	-	-	-	-	-	*Legionella israelensis*	86	NR_036894
GP11	-	-	-	-	-	-	0.6	-	-	-	-	-	-	-	-	-	*Legionella drancourtii*	87	NR_026335
GP12	1.9	0.6	0.6	-	0.6	-	1.2	-	-	-	-	-	-	-	-	-	*Legionella hackeliae*	88	NR_104894
GP13	-	-	0.6	-	-	-	-	-	-	-	-	-	-	-	-	-	*Legionella donaldsonii*	86	Z49724
GP14	0.6	0.6	-	0.6	-	-	-	-	-	-	-	-	-	-	-	-	*Legionella dresdenensis*	87	NR_115062
GP15	-	-	0.6	0.6	-	-	-	-	-	-	-	-	-	-	-	-	*Legionella hackeliae*	89	NR_104894
GP16	-	-	-	-	-	-	-	0.6	-	-	-	-	-	-	-	-	*Legionella hackeliae*	88	NR_104894
GP17	1.9	0.6	4.0	-	0.6	-	0.6	-	-	-	-	-	-	-	-	-	*Natronocella acetinitrilica*	87	NR_044087
GP18	0.6	0.6	0.6	0.6	-	-	-	0.6	-	-	-	-	-	-	-	-	*Legionella hackeliae*	88	NR_104894
GP19	-	-	-	-	-	-	-	0.6	-	-	-	-	-	-	-	-	*Legionella spiritensis*	86	NR_117699
GP20	-	-	0.6	0.6	0.6	-	-	-	-	-	-	-	-	-	-	-	*Legionella hackeliae*	88	NR_104894
GP21	-	-	-	-	-	-	-	-	-	-	-	-	-	-	1.2	-	*Coxiella burnetii*	87	CP000890
GP22	-	-	-	-	-	-	-	0.6	-	-	-	-	-	-	-	-	*Legionella hackeliae*	87	NR_104894
GP23	-	-	-	0.6	-	-	-	-	-	-	-	-	-	-	-	-	*Legionella hackeliae*	88	NR_104894
GP24	-	-	-	-	-	-	-	0.6	-	-	-	-	-	-	-	-	*Legionella drancourtii*	88	NR_026335
GP25	0.6	-	-	-	-	-	-	-	-	-	-	-	-	-	-	-	*Legionella hackeliae*	87	NR_104894
GP26	-	-	-	-	-	0.6	-	-	-	-	-	-	-	-	-	-	*Legionella drancourtii*	86	NR_026335
GP27	-	-	0.6	-	-	-	-	-	-	-	-	-	-	-	-	-	*Legionella hackeliae*	88	NR_104894
GP28	-	0.6	-	-	-	-	-	-	-	-	-	-	-	-	-	-	*Legionella hackeliae*	88	NR_104894
GP29	-	0.6	-	-	-	-	-	-	-	-	-	-	-	-	-	-	*Legionella hackeliae*	86	NR_104894
GP30	-	0.6	0.6	-	-	-	-	-	-	-	-	-	-	-	-	-	*Legionella tunisiensis*	87	NR_109416
GP31	-	-	-	-	-	0.6	-	-	-	-	-	-	-	-	-	-	*Legionella cardiaca*	88	NR_109426
GP32	0.6	-	-	-	-	-	-	-	-	-	-	-	-	-	-	-	*Legionella adelaidensis*	87	NR_044952
GP33	-	-	-	-	-	-	0.6	-	-	-	-	-	-	-	-	-	*Aquicella siphonis*	93	AY359284
GP34	0.6	-	-	-	-	-	0.6	0.6	-	-	-	-	-	-	-	-	*Aquicella siphonis*	92	NR_025764
GP35	-	0.6	-	-	-	-	-	-	-	-	-	-	-	-	-	-	*Aquicella siphonis*	93	AY359284
GP36	-	-	-	-	-	0.6	-	-	-	-	-	-	-	-	-	-	*Aquicella siphonis*	91	AY359284
GP37	10.9	0.6**	0.6**	-**	1.2**	3.4**	1.2**	1.7**	-	-	-	-	-	-	-	-	*Aquicella siphonis*	92	AY359284
GP38	-	-	-	-	-	-	-	-	-	-	-	1.0	-	-	-	-	*Aquicella siphonis*	93	AY359284
GP39	-	-	-	-	0.6	-	-	-	-	-	-	-	-	-	-	-	*Aquicella siphonis*	90	NR_025764
GP40	0.6	-	0.6	-	-	0.6	-	1.1	-	-	-	-	-	-	-	-	*Aquicella siphonis*	92	AY359284
GP41	-	-	-	-	0.6	-	0.6	-	-	-	-	-	-	-	-	-	*Aquicella siphonis*	94	NR_025764
GP42	-	-	-	-	-	0.6	-	-	-	-	-	-	-	-	-	-	*Aquicella siphonis*	93	NR_025764
GP43	-	0.6	-	-	-	0.6	-	-	-	-	-	-	-	-	-	-	*Aquicella siphonis*	93	AY359284
GP44	-	-	-	-	-	0.6	-	-	-	-	-	-	-	1.1	-	-	*Aquicella siphonis*	93	AY359284
GP45	-	-	-	-	-	-	-	0.6	-	-	-	1.0	-	-	-	-	*Aquicella siphonis*	92	NR_025764
GP46	-	-	-	-	-	0.6	-	-	-	-	-	-	-	-	-	-	*Aquicella siphonis*	93	NR_025764
GP47	5.8	13.9*	8.1	11.7	4.1	2.3	3.7	2.8	-	1.9	-	1.0	-	-	-	-	*Aquicella siphonis*	92	NR_025764
GP48	-	-	-	0.6	-	-	-	-	-	-	-	-	-	-	-	-	*Aquicella siphonis*	91	NR_025764
GP49	0.6	-	-	-	0.6	-	-	-	-	-	-	-	-	-	-	-	*Aquicella siphonis*	91	AY359284
GP50	-	-	-	0.6	-	-	1.2	-	-	-	-	-	-	-	-	-	*Aquicella siphonis*	90	AY359284
GP51	0.6	-	-	-	-	-	-	-	-	-	-	-	-	-	-	-	*Nitrosococcus halophilus*	87	NR_114774
GP52	0.6	-	-	-	-	-	-	-	-	-	-	-	-	-	-	-	*Candidatus* Allobeggiatoa halophila	86	EU919200
GP53	-	-	-	-	-	-	0.6	-	-	-	-	-	-	-	-	-	*Legionella tunisiensis*	96	NR_109416
GP54	-	-	-	-	-	-	0.6	-	-	-	-	-	-	-	-	-	*Legionella feeleii*	95	NR_026117
GP55	-	-	-	-	-	-	0.6	-	-	-	-	-	-	-	-	-	*Legionella drozanskii*	95	NR_036803
GP56	-	-	-	-	-	0.6	-	-	-	-	-	-	-	-	-	-	*Legionella massiliensis*	95	NR_109415
GP57	-	-	0.6	-	-	0.6	-	-	-	-	-	-	-	-	-	-	*Legionella maceachernii*	91	JX827101
GP58	-	-	-	-	-	0.6	-	0.6	-	-	-	-	-	-	-	-	*Legionella gresilensis*	93	NR_028744
GP59	-	-	1.2	-	-	-	-	-	-	-	-	-	-	-	-	-	*Legionella drancourtii*	97	NR_026335
GP60	-	0.6	-	-	-	0.6	1.2	-	-	-	-	-	-	-	-	-	*Legionella drancourtii*	98	NR_026335
GP61	-	-	-	-	-	-	-	-	0.7	-	-	-	-	-	-	-	*Legionella longbeachae*	96	AY444741
GP62	-	-	-	-	-	-	0.6	-	-	-	-	-	-	-	-	-	*Legionella lytica*	95	NR_118983
GP63	-	-	-	-	0.6	-	-	-	-	-	-	-	-	-	-	-	*Legionella pneumophila* subsp. *Pneumophila*	94	KF537566
GP64	-	0.6	-	-	-	-	-	-	-	-	-	-	-	-	-	-	*Legionella lytica*	86	NR_118983
GP65	-	-	-	1.2	-	-	-	-	-	-	-	-	-	-	-	-	*Microbulbifer chitinilyticus*	87	NR_112918
GP66	0.6	-	-	0.6	0.6	-	-	-	-	-	-	-	-	-	-	-	*Thiohalomonas denitrificans*	87	NR_044097
GP67	-	1.2	0.6	0.6	-	-	-	-	-	-	-	-	-	-	-	-	*Methylomonas koyamae*	86	NR_113033
GP68	-	-	-	-	-	0.6	-	-	-	-	-	-	-	-	-	-	*Legionella drozanskii*	86	NR_036803
GP69	-	-	-	-	-	-	0.6	-	-	-	-	-	-	-	-	-	*Thiohalomonas denitrificans*	85	EF455919
GP70	-	-	-	-	-	-	0.6	-	-	-	-	-	-	-	-	-	*Rickettsiella tipulae*	86	EU180598
GP71	-	0.6	-	-	-	-	-	-	-	-	-	-	-	-	-	-	*Rickettsiella grylli*	86	U97547
GP72	-	-	-	-	-	-	0.6	-	-	-	-	-	-	-	-	-	*Thiohalomonas denitrificans*	86	NR_044097
GP73	0.6	-	-	-	-	-	-	-	-	-	-	-	-	-	-	-	*Coxiella burnetii*	86	AY342037
GP74	-	-	-	-	-	0.6	-	-	-	-	-	-	-	-	-	-	*Methylococcus capsulatus*	87	KF952440
GP75	-	-	0.6	-	-	-	-	-	-	-	-	-	-	-	-	-	*Coxiella burnetii*	86	CP000890
GP76	0.6	0.6	1.2	-	-	0.6	-	-	-	-	-	-	-	-	-	-	*Ectothiorhodospira variabilis*	87	AM943125
GP77	-	-	-	0.6	-	-	-	-	-	-	-	-	-	-	-	-	*Coxiella burnetii*	86	CP000890
GP78	-	0.6	-	-	-	-	-	-	-	-	-	-	-	-	-	-	*Nitrosococcus oceani*	87	NR_074330
GP79	-	-	-	0.6	-	-	-	-	-	-	-	-	-	-	-	-	*Nitrosococcus halophilus*	85	NR_114774
GP80	-	-	-	-	-	-	-	-	-	-	-	-	-	-	-	1.1	*Coxiella cheraxi*	92	NR_116014
GP81	0.6	0.6	-	-	-	-	-	-	-	-	-	-	-	-	-	-	*Ectothiorhodosinus mongolicus*	87	NR_042831
GP82	-	-	-	0.6	-	-	-	-	-	-	-	-	-	-	-	-	*Coxiella burnetii*	86	NR_074154
GP83	-	-	-	0.6	-	-	-	-	-	-	-	-	-	-	-	-	*Ectothiorhodosinus mongolicus*	87	NR_042831
GP84	-	-	-	-	-	-	0.6	-	-	-	-	-	-	-	-	-	*Nitrosococcus halophilus*	87	NR_074790
GP85	-	-	0.6	-	-	-	-	-	-	-	-	-	-	-	-	-	*Marinobacterium nitratireducens*	87	HM032811
GP86	-	-	-	0.6	-	-	-	0.6	-	-	-	-	-	-	-	-	*Moraxella lacunata*	87	AF005161
GP87	0.6	-	1.2	-	-	-	-	-	-	-	-	-	-	-	-	-	*Ectothiorhodosinus mongolicus*	85	NR_042831
GP88	-	-	-	-	-	-	0.6	-	-	-	-	-	-	-	-	-	*Acidiferrobacter thiooxydans*	86	NR_114629
GP89	0.6	0.6	-	-	-	0.6	0.6	-	-	-	-	-	-	-	-	-	*Ectothiorhodosinus mongolicus*	87	NR_042831
GP90	-	-	-	-	-	-	0.6	-	-	-	-	1.0	-	1.1	-	-	*Legionella hackeliae*	86	NR_104894
GP91	-	-	1.2	-	-	0.6	-	-	-	-	-	-	1.4	-	-	-	*Methylobacter whittenburyi*	86	NR_029242
GP92	-	-	-	-	-	-	0.6	-	-	-	-	-	-	-	-	-	*Marinomonas balearica*	87	NR_116237

GP93	0.6	-	-	-	-	-	-	-	-	-	-	-	-	-	-	-	*Marinobacter maritimus*	85	JQ825265
GP94	-	-	-	-	-	-	0.6	-	-	-	-	-	-	-	-	-	*Legionella brunensis*	88	NR_026520
GP95	-	-	-	-	-	-	0.6	-	-	-	-	-	-	-	-	-	*Methylomicrobium album*	87	NR_029244
GP96	-	0.6	0.6	-	-	-	-	-	-	-	-	-	-	-	-	-	*Coxiella burnetii*	88	NR_074154
GP97	-	-	-	-	-	-	-	0.6	-	-	-	-	-	-	-	-	*Thiorhodospira sibirica*	88	HQ877088
GP98	-	-	0.6	-	-	-	-	-	-	-	-	-	-	-	-	-	*Natronocella acetinitrilica*	87	NR_044087
GP99	-	-	-	-	0.6	-	-	-	-	-	-	-	-	-	-	-	*Natronocella acetinitrilica*	86	NR_044087
GP100	-	-	0.6	-	-	-	-	-	-	-	-	-	-	-	-	-	*Methylobacter whittenburyi*	90	NR_029242
GP101	-	-	-	-	-	0.6	-	-	-	-	-	-	-	-	-	-	*Methylobacter whittenburyi*	89	NR_029242
GP102	-	-	-	-	-	-	0.6	-	-	-	-	-	-	-	-	-	*Methylococcus capsulatus*	90	X72771
GP103	-	1.8	4.0	1.2	-	0.6	-	0.6	-	-	-	1.0	-	-	-	-	*Enterobacter amnigenus*	99	HF585042
GP104	-	-	-	1.2	-	-	-	-	-	-	-	-	-	-	-	-	*Lelliottia amnigena*	98	HF562865
GP105	-	-	-	-	1.2	0.6	-	2.3	-	-	-	-	-	-	-	-	*Pectobacterium carotovorum* subsp. *carotovorur*	100	AB680143
GP106	-	-	-	3.1	2.4	-	-	2.3	-	-	-	-	-	-	-	-	*Pantoea dispersa*	100	KC510017
GP107	-	0.6	-	-	0.6	-	0.6	-	-	-	-	-	-	-	-	-	*Serratia liquefaciens*	99	CP006252
GP108	-	-	0.6	3.1	1.2	-	0.6	-	-	-	-	-	-	-	-	-	*Pantoea agglomerans*	100	KJ210675
GP109	-	-	-	-	-	-	-	-	-	1.9	-	-	-	-	-	-	*Pseudomonas putida*	97	EU601175
GP110	-	0.6	1.7	1.2	1.2	0.6	0.6	1.1	0.7	3.8	-	-	4.3	-	-	4.2	*Pseudomonas koreensis*	100	KF307692
GP111	-	-	-	0.6	-	-	-	-	-	-	-	-	-	-	-	-	*Pseudomonas abietaniphila*	99	NR_041952
GP112	-	1.8	1.7	0.6	-	0.6	0.6	1.7	-	-	4.7	-	-	-	1.2	-	*Pseudomonas arsenicoxydans*	100	HG424437
GP113	-	-	-	-	-	-	-	0.6	-	-	-	-	-	-	-	-	*Acinetobacter calcoaceticus*	100	KJ534248
GP114	-	-	-	2.5	5.3	0.6	-	-	-	-	-	-	-	-	-	-	*Acinetobacter guillouiae*	100	KF749283
GP115	-	-	-	-	-	-	-	-	-	-	-	-	-	-	-	10.5	*Enhydrobacter aerosaccus*	100	AB931121
GP116	-	0.6	-	-	2.4	-	-	-	-	-	-	-	-	1.1	-	-	*Luteimonas aestuarii*	97	KF876901
GP117	-	0.6	-	-	-	-	-	-	-	-	-	1.0	-	-	-	-	*Lysobacter oligotrophicus*	98	NR_114317
GP118	-	-	0.6	-	0.6	-	-	0.6	4.4	-	-	-	1.4	-	-	-	*Stenotrophomonas maltophilia*	100	KJ472806
GP119	-	-	-	-	-	-	-	0.6	-	-	-	-	-	-	-	-	*Dokdonella fugitiva*	97	NR_042397
GP120	-	-	-	1.2	-	-	-	-	-	-	-	-	-	-	-	-	*Rhodanobacter umsongensis*	98	NR_108435
GP121	-	-	-	-	-	-	-	0.6	-	-	-	-	-	-	-	-	*Luteibacter yeojuensis*	100	FN796854
GP122	0.6	1.8	-	-	1.8	-	-	1.1	-	-	-	-	-	-	-	-	*Rhodanobacter thiooxydans*	99	HG794332
GP123	0.6	1.2	-	-	-	0.6	1.2	-	-	-	-	-	-	-	-	-	*Gynumella flava*	97	EU883642
GP124	-	-	-	-	-	-	-	-	-	-	-	-	-	-	1.2	-	*Candidatus* Odyssella thessalonicensis	85	AF069496
DP1	0.6	-	-	-	-	-	-	-	-	-	-	-	-	-	-	-	*Sandaracinus amylolyticus*	84	NR_118001
DP2	-	-	-	-	-	-	-	-	-	-	-	-	1.4	-	-	-	*Anaeromyxobacter dehalogenans*	82	KC921178
DP3	-	-	-	0.6	-	-	-	-	-	-	-	-	-	-	-	-	*Candidatus* Lariskella arthropodarum	90	JQ726744
DP4	-	-	-	-	-	-	-	-	-	1.9	-	-	-	-	-	-	*Stigmatella koreensis*	80	EF112185
DP5	-	-	-	-	-	-	-	-	-	-	-	1.0	-	-	-	-	*Desulfuromonas acetexigens*	81	NR_044770
DP6	-	-	-	-	-	0.6	-	-	-	-	-	-	-	-	-	-	*Geobacter lovleyi SZ*	81	NR_115337
DP7	5.1	0.6	5.8	1.2	1.2	1.1	6.7	5.6	-	-	-	-	-	-	-	-	*Corallococcus macrosporus*	95	KF267729
DP8	0.6	-	-	-	-	-	-	-	-	-	-	-	-	-	-	-	*Desulfuromonas acetoxidans*	81	NR_121678
DP9	-	-	-	-	-	-	0.6	-	-	-	-	-	-	-	-	-	*Geobacter bemidjiensis*	84	KF952444
DP10	-	-	-	-	-	-	0.6	-	-	-	-	-	-	-	-	-	*Bdellovibrio bacteriovorus*	89	EU884925
DP11	-	-	-	-	-	-	0.6	-	-	-	4.7	-	-	1.1	-	-	*Leptolyngbya nostocorum*	83	JQ070063
FM1	-	-	-	-	-	-	-	-	-	-	-	-	-	1.1	1.2	-	*Clostridium puniceum*	99	NR_026105
FM2	-	-	-	-	-	-	-	-	-	-	2.3	-	-	-	-	-	*Clostridium bowmanii*	99	NR_114879
FM3	-	-	-	-	-	-	-	-	-	-	2.3	-	-	-	-	-	*Clostridium intestinale*	99	AM158323
FM4	-	-	-	-	-	-	-	-	-	-	-	-	1.4	-	-	-	*Clostridium thermocellum*	92	HG917929
FM5	-	-	-	-	-	-	-	-	-	-	-	1.0	-	-	-	-	*Parasporobacterium paucivorans*	96	NR_025390
FM6	-	-	-	-	-	-	-	-	-	-	-	1.0	1.4	-	-	-	*Paenibacillus castaneae*	99	HG974509
FM7	0.6	-	-	-	-	-	-	-	-	-	-	-	-	-	-	-	*Paenibacillus telluris*	99	EU934091
FM8	-	-	-	0.6	-	-	-	-	-	-	-	-	-	-	-	-	*Paenibacillus agarexedens*	99	KC355292
FM9	-	-	-	-	-	0.6	-	-	-	-	-	-	-	-	-	-	*Paenibacillus agaridevorans*	95	NR_025490
FM10	-	3.6	-	-	1.8	-	0.6	-	-	-	-	-	-	-	-	-	*Paenibacillus turicensis*	99	HM069128
FM11	-	0.6	-	-	-	-	-	-	-	-	-	-	-	-	-	-	*Paenibacillus xylanexedens*	98	JX035957
FM12	5.8	1.2*	2.9	6.8	8.2	5.7	2.4	4.5	-	-	-	-	4.3	-	-	-	*Paenibacillus pabuli*	100	KC550588
FM13	-	-	-	0.6	-	-	0.6	-	-	-	-	-	-	-	-	-	*Paenibacillus massiliensis*	99	KF515733
FM14	-	-	-	-	-	-	0.6	-	-	-	-	-	-	-	-	-	*Paenibacillus donghaensis*	99	NR_115947
FM15	-	-	-	-	-	-	-	0.6	-	-	-	-	-	-	-	1.1	*Paenibacillus caespitis*	99	AM745263
FM16	0.6	-	-	-	-	-	-	-	-	-	-	1.0	-	-	-	-	*Paenibacillus terrigena*	91	GQ284528
FM17	-	-	-	-	-	-	-	-	-	-	-	-	1.4	-	-	-	*Paenibacillus terrigena*	91	GQ284528
FM18	-	-	-	-	-	-	-	0.6	-	-	-	1.0	-	-	-	-	*Paenibacillus terrigena*	91	FJ174603
FM19	-	-	-	-	-	-	-	-	-	-	-	-	1.4	-	-	-	*Paenibacillus terrigena*	91	GQ284528
FM20	8.3	5.5	4.6	6.8	2.9	1.1	7.3	4.5	1.5	3.8	-	2.1	1.4	3.3	-	1.1	*Paenibacillus alginolyticus*	99	NR_040893
FM21	2.6	1.2	0.6	0.6	-	1.1	-	0.6	-	-	-	-	-	-	-	-	*Paenibacillus pectinilyticus*	99	JX401499
FM22	-	-	-	-	-	-	-	-	-	-	-	-	1.4	-	-	1.1	*Paenibacillus aestuarii*	96	NR_116365
FM23	-	-	-	-	-	-	-	-	0.7	-	-	-	1.4	-	-	-	*Paenibacillus alginolyticus*	97	JF496385
FM24	-	-	-	-	-	-	-	-	0.7	-	-	-	1.4	-	-	-	*Paenibacillus aestuarii*	94	NR_116365
FM25	-	-	-	-	-	-	-	-	-	-	-	-	-	1.1	-	1.1	*Paenibacillus pocheonensis*	94	NR_112565
FM26	-	-	-	-	-	-	-	-	-	-	-	-	1.4	-	-	-	*Paenibacillus chartarius*	94	NR_108483
FM27	1.3	-	-	0.6	-	-	0.6	-	-	-	-	-	-	-	-	-	*Paenibacillus chartarius*	94	NR_108483
FM28	-	-	-	0.6	-	-	-	-	-	-	-	-	-	-	-	-	*Paenibacillus chartarius*	94	NR_108483
FM29	-	-	-	-	-	-	-	-	-	-	-	1.0	-	-	-	-	*Cohnella phaseoli*	99	NR_044374
FM30	-	-	-	-	-	-	-	-	-	-	-	2.1	-	-	-	-	*Cohnella boryungensis*	96	NR_108985
FM31	-	-	-	-	-	-	-	-	0.7	-	-	-	-	-	-	-	*Cohnella luojiensis*	94	HF585307
FM32	-	-	-	-	-	-	-	-	-	1.9	-	-	-	-	-	-	*Bacillus firmus*	99	EF636895
FM33	-	-	-	-	-	-	-	-	-	-	-	-	-	1.1	-	-	*Bacillus longiquaesitum*	99	AM747042
FM34	-	-	-	-	-	-	-	-	-	-	-	1.0	-	-	-	-	*Bacillus circulans*	99	JQ337953
FM35	-	-	-	-	-	-	-	-	-	-	-	-	-	1.1	-	-	*Bacillus bataviensis*	99	EU977785
FM36	-	-	-	-	-	-	-	-	0.7	-	-	-	-	-	-	6.3	*Bacillus megaterium*	100	KJ789855
FM37	-	-	-	-	-	-	-	-	-	1.9	-	-	-	-	-	1.1	*Bacillus niabensis*	99	KC494318
FM38	-	-	-	-	-	-	0.6	-	-	-	-	-	-	-	-	-	*Bacillus aerophilus*	100	KJ616371
FM39	-	-	-	-	-	-	-	-	-	-	-	1.0	-	-	-	-	*Bacillus niabensis*	95	FR877767
FM40	-	-	-	-	-	-	-	-	-	-	-	-	1.4	-	-	-	*Bacillus shackletonii*	98	HF584959
FM41	2.6	5.5	3.5	2.5	-	1.7	5.5	5.1	31.1	26.4	60.5*	36.1	27.5	16.3*	7.4**	25.3	*Bacillus halmapalus*	100	FJ188305
FM42	-	-	-	-	-	-	0.6	-	-	-	-	2.1	2.9	-	-	-	*Staphylococcus epidermidis*	100	KJ438691
FM43	-	-	-	-	-	-	-	-	-	1.9	-	-	-	-	-	-	*Psychrobacillus psychrodurans*	99	JX840983
FM44	-	-	-	-	-	-	-	-	-	-	-	-	-	-	-	1.1	*Sporosarcina globispora*	97	KF956659
FM45	-	-	-	-	-	-	-	-	-	-	-	1.0	-	-	-	-	*Lysinibacillus parviboronicapiens*	99	KF208472
FM46	-	-	-	-	-	-	-	-	-	-	-	1.0	-	-	-	-	*Bacillus gibsonii*	100	AB617549
FM47	-	-	1.2	-	-	-	-	-	0.7	-	-	2.1	1.4	-	1.2	-	*Bacillus clausii*	99	KJ642606
FM48	-	-	-	-	-	-	-	-	-	-	-	1.0	-	-	-	-	*Ureibacillus thermosphaericus*	99	AY294314
FM49	-	-	-	-	-	-	-	-	-	-	-	-	-	1.1	-	-	*Ureibacillus terrenus*	99	NR_025394
FM50	-	0.6	-	-	-	-	-	-	-	-	-	-	-	-	-	-	*Gemella morbillorum*	99	AB775577
FM51	-	-	-	-	-	-	-	-	-	-	-	1.0	-	-	-	-	*Kroppenstedtia eburnea*	89	NR_117025

FM52	-	-	-	-	-	-	-	-	-	-	-	-	-	1.1	-	-	*Ammoniphilus oxalivorans*	87	HG974521
FM53	-	-	-	-	-	-	-	-	0.7	-	2.3	-	-	-	-	-	*Desulfosporosinus burensis*	97	NR_109421
FM54	-	-	-	-	-	-	-	-	-	-	-	1.0	-	-	-	-	*Pelosinus defluvii*	97	NR_109397
FM55	-	-	-	-	-	-	-	-	-	-	-	1.0	-	-	-	-	*Moorella stamsii*	85	NR_108896
FM56	-	-	-	-	-	-	-	-	1.5	-	-	-	-	-	-	-	*Hydrogenispora ethanolica*	92	AB669474
FM57	-	-	-	-	-	-	-	-	-	-	-	-	-	-	-	1.1	*Hydrogenispora ethanolica*	87	AB669474
FM58	-	-	-	-	-	-	-	-	-	-	2.3	-	-	-	-	1.1	*Pelotomaculum thermopropionicum*	85	NR_074685
FM59	-	-	-	-	-	-	-	-	-	-	-	-	-	-	-	1.1	*Thermohalobacter berrensis*	85	NR_024960
AC1	-	-	-	-	-	-	-	0.6	-	-	-	-	-	-	-	-	*Longispora albida*	95	NR_024800
AC2	-	-	-	-	-	-	-	-	-	-	-	1.0	-	-	-	-	*Actinocorallia aurea*	99	AB006160
AC3	-	-	-	-	-	0.6	-	-	-	-	-	-	-	-	-	-	*Actinomadura scrupuli*	94	NR_116909
AC4	0.6	1.2	-	-	-	-	0.6	-	-	-	-	-	-	-	-	-	*Arthrobacter cryoconiti*	96	NR_108846
AC5	1.3	1.8	1.7	1.9	2.4	1.7	1.2	1.7	0.7	-	-	1.0	-	1.1	1.2	2.1	*Arthrobacter nicotinovorans*	100	KF979130
AC6	-	-	-	-	-	-	-	-	-	-	-	-	-	-	1.2	-	*Rothia amarae*	100	FR682692
AC7	-	-	-	-	-	-	-	0.6	-	-	-	-	-	-	-	-	*Microbacterium oxydans*	99	KJ729148
AC8	-	-	-	-	-	-	-	-	-	-	-	1.0	-	-	-	-	*Microbacterium testaceum*	99	JN585696
AC9	-	-	-	-	-	-	-	0.6	-	-	-	-	-	-	-	-	*Microbacterium xylanilyticum*	100	JF700460
AC10	-	-	-	-	-	-	-	1.1	-	-	-	-	-	-	-	-	*Plantibacter cousiniae*	99	JQ661206
AC11	-	-	-	-	-	0.6	-	-	-	-	-	-	-	-	-	-	*Frigoribacterium faeni*	99	KJ472829
AC12	-	1.2	-	-	0.6	0.6	-	-	-	-	-	1.0	-	-	-	-	*Leifsonia xyli*	99	GU332619
AC13	-	0.6	-	0.6	-	-	-	-	-	-	-	-	-	-	1.2	-	*Streptomyces sanglieri*	99	JF755888
AC14	-	-	-	-	-	-	0.6	-	-	-	-	-	-	-	-	-	*Kitasatospora mediocidica*	97	NR_112444
AC15	-	-	-	-	-	-	-	0.6	-	-	-	-	-	-	-	-	*Streptomyces bingchenggensis*	99	NR_074824
AC16	3.8	1.2	2.3	-	4.1	0.6	1.2	0.6	-	-	2.3	-	1.4	1.1	-	-	*Streptomyces rishiriensis*	99	JQ342919
AC17	0.6	-	-	-	-	-	-	-	-	-	-	-	-	-	-	-	*Streptomyces indigoferus*	92	JX840953
AC18	-	-	-	-	-	-	-	0.6	-	-	-	-	-	-	-	-	*Mycobacterium smegmatis*	97	CP003078
AC19	0.6	-	-	-	-	-	-	-	-	-	-	-	-	-	-	-	*Mycobacterium pyrenivorans*	99	JX841025
AC20	1.9	-	0.6	-	-	-	-	-	-	1.9	-	5.2	-	-	-	1.1	*Mycobacterium llatzerense*	100	HG810917
AC21	-	-	-	-	-	-	-	-	-	-	-	-	-	1.1	-	-	*Mycobacterium frederiksbergense*	99	HG941823
AC22	-	-	-	-	-	-	-	-	-	-	-	-	-	-	1.2	-	*Mycobacterium llatzerense*	91	HG810917
AC23	-	-	-	-	-	1.1	-	-	-	-	-	-	2.9	-	-	-	*Smaragdicoccus niigatensis*	99	NR_041353
AC24	1.9	3.0	2.9	-	3.5	2.9	1.2	1.7	1.5	1.9	-	-	1.4	12.0**	8.6**	20.0**	*Rhodococcus baikonurensis*	100	KJ571097
AC25	-	-	-	0.6	-	-	0.6	-	-	-	-	-	1.4	-	-	-	*Nocardia vinacea*	99	HG941915
AC26	-	-	-	-	-	-	-	-	-	-	-	-	1.4	-	-	-	*Nocardia inohanensis*	99	AJ619769
AC27	-	-	-	-	0.6	-	-	-	-	-	-	-	-	-	-	-	*Catenulispora graminis*	97	NR_109018
AC28	-	-	-	-	-	-	-	0.6	-	-	-	-	-	-	-	-	*Catenulispora graminis*	92	NR_109018
AC29	-	-	-	-	0.6	-	-	0.6	-	-	-	-	-	-	-	-	*Catenulispora cavernae*	99	FM998835
AC30	0.6	-	-	-	-	-	-	-	-	-	-	-	-	-	-	-	*Aeromicrobium ginsengisoli*	100	JX841017
AC31	-	-	-	-	-	-	-	-	0.7	-	-	-	-	-	-	-	*Aeromicrobium kwangyangensis*	99	EF693740
AC32	-	-	-	-	-	0.6	-	-	-	-	-	-	-	-	-	-	*Nocardioides jensenii*	97	NR_044981
AC33	-	-	-	-	-	-	0.6	-	-	-	-	-	-	-	-	-	*Nocardioides halotolerans*	98	NR_044234
AC34	-	-	0.6	-	-	-	-	-	-	-	-	-	-	-	-	-	*Propionibacterium acnes*	100	CP006032
AC35	-	-	-	-	-	-	-	-	-	-	-	1.0	-	-	-	-	*Iamia majanohamensis*	90	NR_041634
AC36	-	-	0.6	-	-	-	-	-	-	-	-	-	-	-	-	-	*Gaiella occulta*	92	NR_118138
AC37	-	-	-	-	-	-	-	-	-	-	-	1.0	-	-	-	-	*Patulibacter medicamentivorans*	84	NR_121691
AC38	-	-	-	-	-	-	-	0.6	-	-	-	-	-	-	-	-	*Conexibacter arvalis*	91	AB597951
PL1	-	-	-	-	-	0.6	-	0.6	-	-	-	-	-	-	-	-	*Telmatocola sphagniphila*	88	NR_118328
PL2	0.6	-	-	-	-	-	-	-	-	-	-	-	-	-	-	-	*Telmatocola sphagniphila*	88	NR_118328
PL3	1.3	0.6	0.6	0.6	-	1.1	0.6	-	-	-	-	-	-	2.2	2.5	-	*Telmatocola sphagniphila*	89	NR_118328
PL4	-	-	-	-	0.6	-	-	-	-	-	-	-	-	-	-	-	*Telmatocola sphagniphila*	85	NR_118328
PL5	-	-	-	-	-	0.6	-	-	-	-	-	-	-	-	-	-	*Gemmata obscuriglobus*	81	X85248
PL6	-	-	-	-	-	0.6	-	-	-	-	-	-	-	-	-	-	*Gemmata obscuriglobus*	96	X81957
PL7	-	-	-	-	-	0.6	-	-	-	-	-	-	-	-	-	-	*Gemmata obscuriglobus*	89	X85248
PL8	-	-	-	-	-	0.6	-	-	-	-	-	-	-	-	-	-	*Gemmata obscuriglobus*	86	NR_114712
PL9	-	-	-	0.6	-	-	-	-	-	-	-	-	-	-	-	-	*Gemmata obscuriglobus*	82	X85248
PL10	-	-	-	-	-	-	0.6	-	-	-	-	-	-	-	-	-	*Gemmata obscuriglobus*	80	NR_114712
PL11	-	-	-	-	-	-	0.6	-	-	-	-	-	-	-	-	-	*Gemmata obscuriglobus*	82	X85248
PL12	-	-	-	-	-	0.6	-	-	-	-	-	-	-	-	-	-	*Gemmata obscuriglobus*	83	X81957
PL13	-	-	0.6	-	-	-	-	-	-	-	-	-	-	-	-	-	*Planctomyces limnophilus*	80	NR_074670
PL14	-	-	-	-	0.6	-	-	-	-	-	-	-	-	-	-	-	*Nostocoida limicola* III	95	AF244749
PL15	-	-	-	-	-	-	0.6	-	-	-	-	-	-	-	-	-	*Singulisphaera mucilaginosa*	95	HM748856
PL16	-	-	1.2	0.6	1.2	0.6	-	-	-	-	-	-	-	1.1	-	-	*Singulisphaera acidiphila*	98	NR_102439
PL17	-	-	-	-	-	-	-	-	-	-	-	1.0	-	-	-	-	*Aquisphaera giovannonii*	94	NR_122081
PL18	-	-	-	-	-	-	0.6	-	-	-	-	-	-	-	-	-	*Nostocoida limicola III*	92	AF244749
PL19	-	-	-	-	-	-	-	1.1	-	-	-	-	-	-	-	-	*Singulisphaera rosea*	88	NR_116969
PL20	-	-	-	0.6	-	-	-	-	-	-	-	-	-	-	-	-	*Schlesneria paludicola*	87	NR_042466
PL21	0.6	-	-	-	-	-	-	-	-	-	-	-	-	-	-	-	*Planctomyces maris*	88	NR_025327
PL22	0.6	-	-	-	-	-	-	-	-	-	-	-	-	-	-	-	*Planctomyces maris*	86	NR_025327
PL23	0.6	-	-	-	-	-	-	-	-	-	-	-	-	-	-	-	*Planctomyces limnophilus*	86	NR_074670
PL24	-	-	-	-	0.6	-	-	-	-	-	-	-	-	-	-	-	*Planctomyces maris*	84	NR_025327
PL25	-	-	-	-	-	2.3	0.6	-	-	-	-	-	-	-	-	-	*Schlesneria paludicola*	99	NR_042466
PL26	-	0.6	-	-	-	0.6	-	-	-	-	-	-	-	-	-	-	*Schlesneria paludicola*	97	NR_042466
PL27	1.3	0.6	0.6	-	-	1.1	1.2	-	-	-	-	-	-	-	-	-	*Schlesneria paludicola*	94	NR_042466
PL28	-	-	-	-	0.6	-	-	-	-	-	-	-	-	-	-	-	*Planctomyces maris*	88	NR_025327
PL29	-	0.6	0.6	-	1.2	0.6	-	-	-	-	-	-	-	-	-	-	*Planctomyces limnophilus*	84	NR_074670
PL30	-	-	-	-	-	-	0.6	-	-	-	-	-	-	-	-	-	*Candidatus* Anammoximicrobium moscowi	86	KC467065
PL31	-	-	-	-	-	0.6	-	-	-	-	-	-	-	-	-	-	*Bythopirellula goksoyri*	85	NR_118636
PL32	-	-	-	-	-	-	-	0.6	-	-	-	-	-	-	-	-	*Chthoniobacter flavus*	90	NR_115225
PL33	-	-	-	-	-	0.6	-	0.6	-	-	-	-	-	-	-	-	*Chthoniobacter flavus*	92	NR_115225
PL34	0.6	-	-	-	-	-	-	-	-	-	-	-	-	-	-	-	*Chthoniobacter flavus*	92	NR_115225
PL35	-	-	-	-	-	0.6	-	-	-	-	-	-	-	-	-	-	*Chthoniobacter flavus*	90	NR_115225
PL36	-	-	-	-	-	-	-	-	-	-	-	-	-	1.1	-	-	*Chthoniobacter flavus*	90	NR_115225
PL37	-	-	-	-	1.2	-	-	0.6	-	-	-	-	-	-	-	-	*Chthoniobacter flavus*	94	NR_115225
PL38	-	-	-	-	-	1.7	0.6	-	-	-	-	-	-	1.1	-	-	*Chthoniobacter flavus*	96	NR_115225
PL39	-	-	-	-	0.6	-	-	-	-	-	-	-	-	-	-	-	*Chthoniobacter flavus*	89	NR_115225
PL40	-	0.6	-	-	-	-	-	-	-	-	-	-	-	-	-	-	*Candidatus* Xiphinematobacter brevicolli	86	AF217462
PL41	-	0.6	-	-	-	-	-	-	-	-	-	-	-	-	-	-	*Candidatus* Xiphinematobacter brevicolli	90	AF217462
PL42	-	-	-	-	-	-	0.6	-	-	-	-	-	-	-	-	-	*Candidatus* Xiphinematobacter brevicolli	89	AF217462
PL43	-	-	-	-	-	-	-	0.6	-	-	-	-	-	-	-	-	*Chthoniobacter flavus*	90	NR_115225
PL44	-	-	-	-	-	1.7	0.6	0.6	-	-	-	-	-	-	-	-	*Chthoniobacter flavus*	87	NR_115225
PL45	-	-	-	-	-	-	0.6	-	-	-	-	-	-	-	-	-	*Candidatus* Xiphinematobacter brevicolli	83	AF217462
PL46	-	-	-	-	0.6	-	-	-	-	-	-	-	-	-	-	-	*Roseimicrobium gellanilyticum*	99	NR_113148
PL47	-	-	-	-	1.2	-	-	-	-	-	-	-	-	-	-	-	*Prosthecobacter fluviatilis*	97	NR_041608
PL48	-	-	-	-	-	0.6	-	-	-	-	-	-	-	-	-	-	*Luteolibacter pohnpeiensis*	99	NR_041625

PL49	-	-	0.6	-	-	0.0	1.2	0.6	-	-	-	-	-	-	-	-	*Coraliomargarita akajimensis*	88	NR_074901
PL50	0.6	0.6	0.6	-	-	0.6	-	0.6	-	-	-	-	-	-	-	-	*Coraliomargarita akajimensis*	88	NR_074901
PL51	-	-	-	-	-	0.6	-	-	-	-	-	-	-	-	-	-	*Gemmatimonas aurantiaca*	87	NR_074708
PL52	-	0.6	-	-	-	-	-	-	-	-	-	-	-	-	-	-	*Thermodesulfobium narugense*	79	NR_074466
PL53	0.6	0.6	-	-	-	-	-	-	-	-	-	-	-	-	-	-	*Desulfonatronum cooperativum*	77	NR_043143
PL54	-	-	-	-	-	-	0.6	-	-	-	-	-	-	-	-	-	*Moorella thermoacetica*	78	NR_043076
PL55	0.6	-	0.6	-	-	-	-	-	-	-	-	-	-	-	-	-	*Desulfatibacillum alkenivorans*	77	NR_074962
PL56	-	-	-	-	-	-	-	0.6	-	-	-	-	-	-	-	-	*Desulfofaba fastidiosa*	78	NR_025746
PL57	-	-	-	-	-	-	-	-	0.7	-	-	-	-	1.1	-	-	*Desulfopila inferna*	78	NR_115066
PL58	0.6	-	-	-	-	-	-	-	-	-	-	-	-	-	-	-	*Stenotrophomonas maltophilia*	78	EF620453
PL59	-	-	-	-	-	-	0.6	-	-	-	-	-	-	-	-	-	*Desulfopila inferna*	76	NR_115066
PL60	-	-	0.6	-	-	-	-	-	-	-	-	-	-	-	-	-	*Desulfonatronum lacustre*	80	NR_041848
BA1	-	-	-	-	1.2	-	-	-	-	-	-	-	-	-	-	-	*Flavobacterium banpakuense*	97	NR_117276
BA2	-	-	-	-	0.6	-	-	-	-	-	-	-	-	-	-	-	*Flavobacterium frigidimaris*	99	KF981555
CH1	-	-	-	0.6	-	-	-	-	-	-	-	-	-	-	-	-	*Thermosporothrix hazakensis*	87	NR_112915
CH2	0.6	-	-	-	-	-	-	-	-	-	-	-	-	-	-	-	*Ktedonobacter racemifer*	89	NR_112949
UB1	-	-	-	-	-	-	-	-	-	-	-	2.1	-	-	-	-	*Desulfotomaculum solfataricum*	84	NR_036846
UB2	-	-	-	0.6	0.6	-	-	-	-	-	-	-	-	-	-	-	*Heliorestis daurensis*	82	NR_028721
UB3	-	-	-	-	-	0.6	-	-	-	-	-	-	-	-	-	-	*Sphaerobacter thermophilus*	82	CP001824
UB4	-	-	-	-	-	0.6	-	-	-	-	-	-	-	-	-	-	*Aciditerrimonas ferrireducens*	81	NR_112972

[a]R, M and S stand for phenotypes of potato 8 genotypes which are resistant, medium resistant and susceptible to potato scab diseases, respectively. R1, R2, R3, M1, M2, S1, S2 and S3 are designated for clone libraries of 8 potato genotypes (Yukirasha, 02005-10, Snow March, Star Rubi, Snowden, Irish Cobbler, Toyoshiro, and Piruka, respectively). CD stands for the isolate collections derived from roots or tubers of cultivar "Yukirasha".

* and ** indicate statistical significance at the 5% and 1% levels ($P < 0.05$ and $P < 0.01$), respectively, based on the results of Fisher's exact test between R1 and other libraries.

Supplementary Table 3A. Statistical significance for the abundance of the genus *Aquicella* among roots of 8 genotypes of potato plants

	R2[a]	R3	M1	M2	S1	S2	S3
R1	-	*	-	**	*	**	**
R2		-	-	*	-	*	**
R3			-	-	-	-	-
M1				-	-	-	-
M2					-	-	-
S1						-	-
S2							-

Supplementary Table 3B. Statistical significance for the abundance of the genus *Rhodococcus* among tubers of 8 genotypes of potato plants

	R2[a]	R3	M1	M2	S1	S2	S3
R1	-	-	-	-	**	**	**
R2		-	-	-	*	-	**
R3			-	-	*	-	**
M1				-	**	**	**
M2					*	-	**
S1						-	-
S2							*

* and ** indicate statistical significance at the 5% and 1% levels ($P < 0.05$ and $P < 0.01$), respectively, calculated with the Library Compare

[a]R, M and S stand for phenotypes of potato 8 genotypes which are resistant, medium resistant and susceptible to potato scab diseases, respectively. R1, R2, R3, M1, M2, S1, S2 and S3 are designated for clone libraries of 8 potato genotypes (Yukirasha, 02005-10, Snow March, Star Ruby, Snowden, Irish Cobbler, Toyoshiro, and Piruka,

Kobayashi et al. 2015

Supplementary Fig. 1

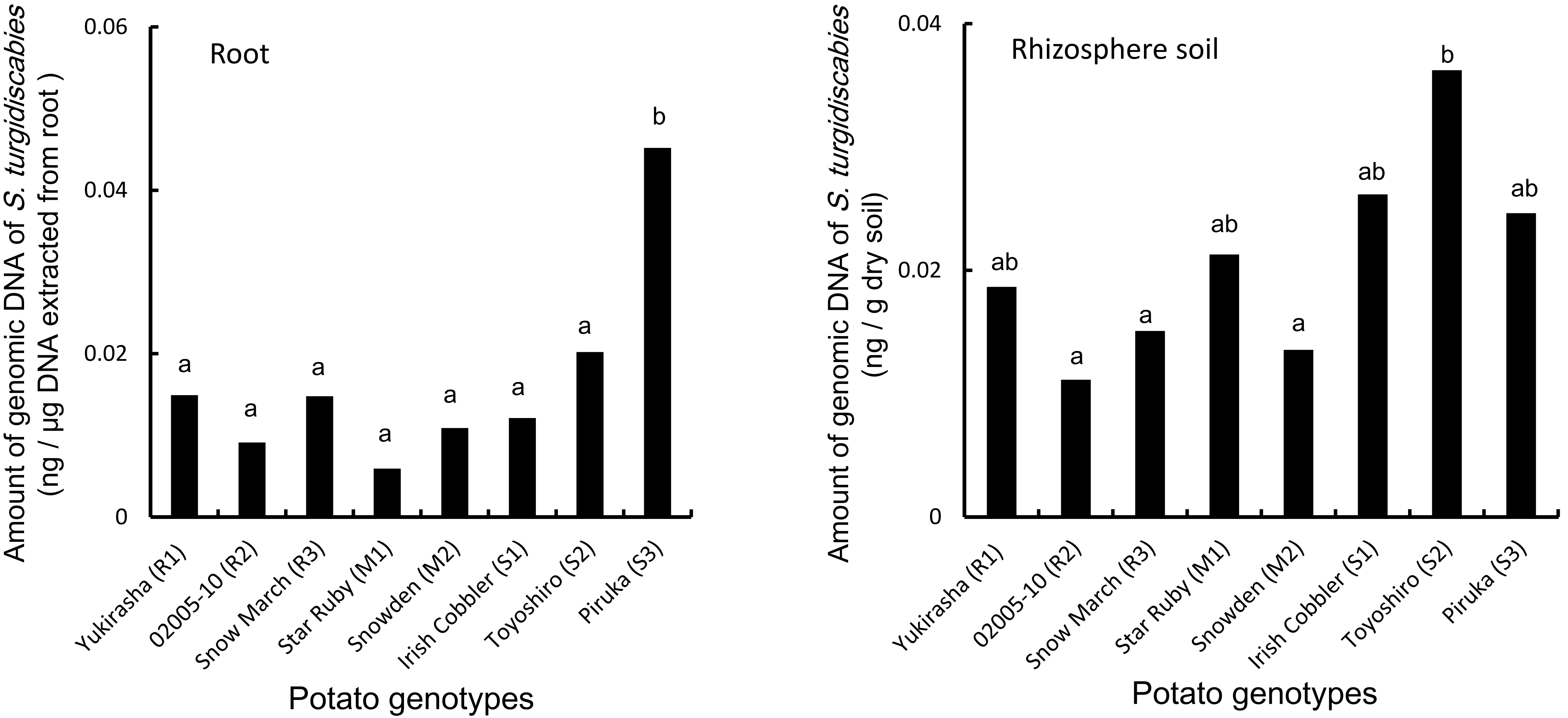

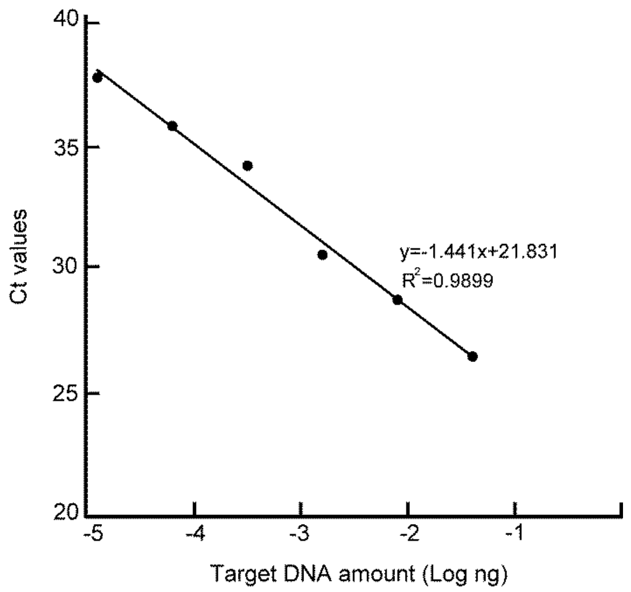
40
35
30
25
20
Ct values
y=-1.441x+21.831
R2=0.9899
-5
-4
-3
-2
-1
Target DNA amount (Log ng)

Fig.S2
